# A double-blind, randomised, placebo-controlled study of roxithromycin and doxycycline combination, roxithromycin alone, or matching placebo for 12 weeks in adults with frequent exacerbations of chronic obstructive pulmonary disease

**DOI:** 10.1186/s12952-015-0034-8

**Published:** 2015-09-07

**Authors:** Eskandarain Shafuddin, Graham D. Mills, Mark D. Holmes, Phillippa J. Poole, Peter R. Mullins, Peter N. Black

**Affiliations:** Respiratory Research Unit, Department of Respiratory Medicine, Waikato Hospital, Hamilton, New Zealand; Respiratory Clinical Trials Unit, Royal Adelaide Hospital, Adelaide, SA Australia; Faculty of Medical and Health Sciences, The University of Auckland and Auckland Hospital, Auckland, New Zealand; Department of Statistics, The University of Auckland, Auckland, New Zealand

## Abstract

**Background:**

Azithromycin prophylaxis has been shown to reduce COPD exacerbations but there is poor evidence for other antibiotics. We compared exacerbation rates in COPD patients with a history of frequent exacerbations (at least three moderate or severe COPD exacerbations in the past two years) during a 12-week treatment course and over a subsequent 48-week follow up period.

**Results:**

292 patients were randomised to one of three treatments for 12 weeks: roxithromycin 300 mg daily and doxycycline 100 mg daily (n = 101); roxithromycin 300 mg daily (n = 97); or matching placebos (n = 94). There were no differences in the annualised moderate and severe exacerbation rates after treatment with roxithromycin/doxycycline (2.83 (95 % CI 2.37-3.40)) or roxithromycin only (2.69 (2.26-3.21)) compared to placebo (2.5 (2.08-3.03)) (p = 0.352 and p = 0.5832 respectively). Furthermore, there were no differences in the annualised exacerbation rates during 12-week treatment with roxithromycin/doxycycline (1.64 (95 % CI 1.17-2.30)), roxithromycin only (1.75 (1.24-2.41)) or placebo (2.23 (1.68-3.03)) (p = 0.1709 and p = 0.2545 respectively). There were also no significant differences between groups for spirometry or quality of life scores over either the 12-week treatment or 48-week post-treatment periods. Both active treatments were associated with nausea but otherwise adverse events were comparable among treatment groups.

**Conclusions:**

Twelve-weeks of prophylaxis with roxithromycin/doxycycline combination or roxithromycin alone did not reduce COPD exacerbations in patients with history of frequent exacerbations. These findings do not support the use of these antibiotics to prevent exacerbations in COPD patients.

**Electronic supplementary material:**

The online version of this article (doi:10.1186/s12952-015-0034-8) contains supplementary material, which is available to authorized users.

## Background

Acute exacerbations of chronic obstructive pulmonary disease (COPD) have a major impact on quality of life and are considered to be a cause of lung function decline [1]. The use of prophylactic antibiotics to reduce exacerbations rates has had a long history with clinical trials dating back to the 1950s. However a 2003 meta-analysis of clinical trials of prophylactic antibiotics in chronic bronchitis found no significant reduction in the frequency of exacerbations [2]. Since then, an improved definition and classification of COPD exacerbations has been introduced, along with several clinical trials of azithromycin and a 2013 Cochrane review indicating a significant reduction in the frequency of COPD exacerbations with prophylactic antibiotics [3–5].

Our study assessed the impact of twelve weeks of the macrolide antibiotic roxithromycin (with and without doxycycline) on exacerbation rate and lung function decline in a COPD population with frequent exacerbations prior to study entry. The study was originally designed to test the hypothesis that *Chlamydia pneumoniae* (now *Chlamydophila pneumoniae*) was a pathogenic factor in the aetiology of COPD and that eradication of *C. pneumoniae* infection could reduce exacerbation rates [6, 7]. This hypothesis is now considered unsubstantiated and is no longer believed to be clinically relevant [8–10]. Nevertheless, this study allowed us to address the role of prophylactic antibiotics in reducing COPD exacerbations.

## Results

### Study participants

**Fig. 1:**
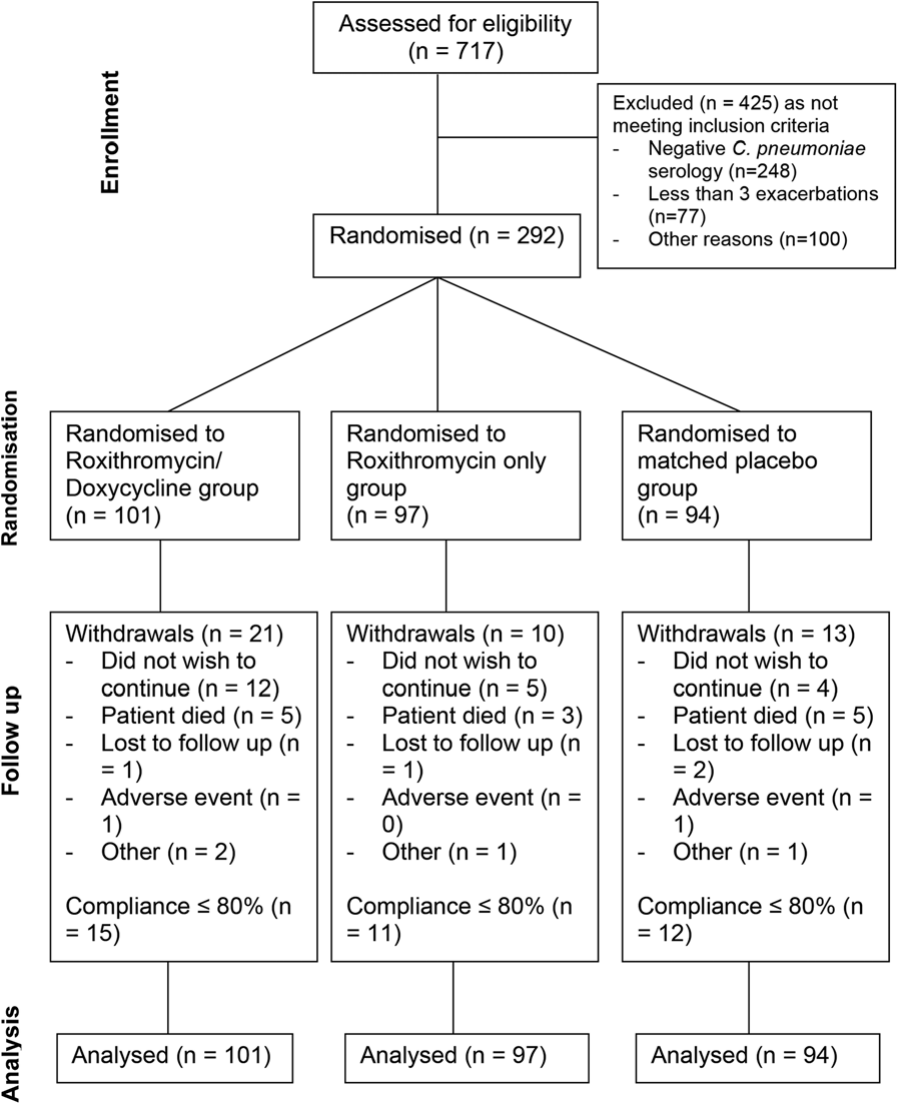
CONSORT diagram of the flow of participants. All randomised participants were included in the analysis. The subjects who had withdrawn from the study medication remained for the full assessment period and were reviewed at the final visit day, as far as possible

The screening, randomisation, withdrawals and protocol deviations are shown in the CONSORT diagram (Fig. 1). Of the 717 patients initially assessed for eligibility, 292 were randomised. Of the 425 excluded subjects, 248 had negative *C. pneumoniae* serology with 77 subjects having fewer than three documented exacerbations over the preceding two-year period. Table 1 compares the characteristics of the 292 included and 425 excluded subjects. Apart from the known difference in *C. pneumoniae* serological status, the major differences were that the included cohort comprised a greater proportion of men (73 % vs. 63 %), worse baseline forced expiratory volume in 1 second (FEV_1_) (mean FEV_1_ predicted 34 % vs. 43 %) and a greater number of exacerbations (5.1 vs. 4.1 exacerbations within the previous 2 years) than the excluded cohort.

**Table 1:** Comparisons of baseline characteristics of the included participants with the excluded participants

	Included	Excluded	*P*-value
Number of patients	292	425	-
Age (year), mean (SD)	67.3 (8.58)	67.9 (9.5)	0.3564
Male, n (%)	214 (73.29 %)	268 (63 %)	0.005
Race, n (%)			
New Zealand European	287 (98.29 %)	414 (97.6 %)	0.637
Maori/Pacific Islander	3 (1.03 %)	6 (1.4 %)	
Other	2 (0.68 %)	4 (1 %)	
Height (cm), mean (SD)	169.8 (8.6)	168 (8.8)	0.018
Weight (kg), mean (SD)	72.6 (16.5)	72.6 (16)	0.993
Smoking history, n (%)			
Current smoker	71 (24.32 %)	116 (28 %)	0.272
Ex-smoker	221 (75.68 %)	298 (72 %)	
Average tobacco consumption (pack year), mean (SD)	56.58 (33.3)	53.7 (30)	0.355
Number of previous exacerbations within 2 years, mean (SD)	5.11 (2.4)	4.15 (2.5)	<0.0001
Spirometry			
FEV_1_ (l), mean (SD)	0.935 (0.43)	1.17 (0.67)	0.0001
FEV_1_ (% predicted), mean (SD)	34 (14.8)	43.5 (23)	<0.0001
FVC (l), mean (SD)	2.23 (0.83)	2.28 (0.93)	0.7983
FEV_1_/FVC mean (SD)	42 (10.2)	50 (14.8)	<0.0001

Some data on spirometry and exacerbation rates in the excluded subjects were missing

The baseline characteristics of the study participants who were randomised are summarised in Table 2. The roxithromycin/doxycycline combination group had more current smokers than the other two groups (p < 0.001), with no other statistically significant differences among the treatment groups.

**Table 2:** Baseline characteristics of participants by treatment groups

	Roxithromycin/Doxycycline	Roxithromycin alone	Placebo
Number of patients	101	97	94
Age (year), mean (SD)	65.8 (7.9)	67.6 (7.85)	66.7 (8.7)
Male, n (%)	64 (63.37 %)	83 (85.57 %)	67 (71.28 %)
Race, n (%)			
New Zealand European	99 (98.02 %)	96 (98.97 %)	92 (97.87 %)
Maori/Pacific Islander	1 (0.99 %)	1 (1.03 %)	1 (1.06 %)
Other	1 (0.99 %)	0	1 (1.06 %)
Height (cm), mean (SD)	168.6 (9.3)	171.4 (7.95)	169.5 (8.2)
Weight (kg), mean (SD)	70.85 (14.6)	72.8 (14.8)	74.5 (19.7)
Smoking history, n (%)			
Current smoker	36 (35.64 %)	17 (17.53 %)	18 (19.15 %)
Ex-smoker	65 (64.36 %)	80 (82.47 %)	76 (80.85 %)
Average tobacco consumption (pack year), mean (SD)	57.96 (30.8)	54.36 (39)	57.38 (29.5)
Number of previous exacerbations within 2 years, mean (SD)	5.01 (2.34)	5.39 (2.56)	4.93 (2.29)
Spirometry			
FEV_1_ (l), mean (SD)	0.85 (0.325)	0.965 (0.455)	0.99 (0.49)
FEV_1_ (% predicted), mean (SD)	32.53 (13.55)	33.93 (15.3)	35.8 (15.2)
FVC (l), mean (SD)	2.11 (0.78)	2.33 (0.82)	2.25 (0.9)
FEV_1_/FVC mean (SD)	41.54 (10)	41.5 (10.8)	43.7 (9.9)
CRQ			
Dyspnoea, median (Min-Max)	15 (5 – 28)	15 (7 – 24)	16 (6 – 31)
Fatigue, median (Min-Max)	15 (5 – 25)	16 (5 – 26)	15 (5 – 26)
Emotional function, median (Min-Max)	33 (13 – 47)	33 (15 – 48)	32 (12 – 49)
Mastery, median (Min-Max)	21 (5 – 28)	21 (7 – 28)	19 (7 – 28)
Co-morbidity (n, %)			
Ischaemic heart disease	6 (5.94 %)	11 (11.34 %)	13 (13.83 %)
Hypertension	36 (35.64 %)	39 (40.21 %)	33 (35.11 %)
Abnormal liver function	1 (0.99 %)	1 (1.03 %)	1 (1.06 %)
Gastroesophageal reflux disease	17 (16.83 %)	15 (15.46 %)	18 (19.15 %)

### Primary outcome

#### COPD exacerbations over 48-week post-treatment period

The annualised moderate and severe event rates were 2.83 per patient year [95 % CI 2.37 – 3.40] in the roxithromycin/doxycycline combination group, 2.69 per patient year [95 % CI 2.26 – 3.21] in the roxithromycin only group and 2.50 per patient year [95 % CI 2.08 – 3.03] in the placebo group. There were no statistically significant differences between either of the two active treatment arms and the placebo group (p = 0.352 in the roxithromycin/doxycycline group and p = 0.5832 in the roxithromycin only group) (Table 3). Mean time to the first moderate or severe event following active treatment period was 121 days (SD 113, 87 patients) in the roxithromycin/doxycycline combination group, 140 days (SD 117, 92 patients) in the roxithromycin only group and 147 days (SD 115, 87 patients) in the placebo group, with no statistically significant difference among the groups (log logistic p = 0.254).

**Table 3:** Moderate and severe exacerbations of COPD by treatment groups

	Roxithromycin + Doxycycline (n = 101)		Roxithromycin (n = 97)		Placebo (n = 94)	
Through 48-week period post treatment						
Number of exacerbations	N	% of overall exacerbations	N	% of overall exacerbations	N	% of overall exacerbations
Overall	263		302		261	
Moderate and severe	217	82.51 %	221	73.18 %	193	73.94 %
Event rate/patient year						
Overall	3.43		3.67		3.38	
Moderate and severe	2.83	^a^P = 0.352	2.69	^a^P = 0.5832	2.50	
During 12-week active treatment period						
Number of exacerbations	N	% of overall exacerbations	N	% of overall exacerbations	N	% of overall exacerbations
Overall	52		51		67	
Moderate and severe	37	71.15 %	39	76.47 %	48	71.64 %
Event rate/patient year						
Overall	2.31		2.27		3.14	
Moderate and severe	1.64	^a^P = 0.1709	1.74	^a^P = 0.2545	2.25	
During the first 24-week period post treatment						
Number of exacerbations	N	% of overall exacerbations	N	% of overall exacerbations	N	% of overall exacerbations
Overall	146		150		141	
Moderate and severe	117	80.13 %	108	72 %	103	73.05 %
Event rate/patient year						
Overall	3.72		3.57		3.55	
Moderate and severe	2.98	^a^P = 0.3864	2.57	^a^P = 0.9577	2.59	
During the last 24 week period post treatment						
Number of exacerbations	N	% of overall exacerbations	N	% of overall exacerbations	N	% of overall exacerbations
Overall	117		152		120	
Moderate and severe	100	85.47 %	113	74.34 %	90	75 %
Event rate/patient year						
Overall	3.12		3.78		3.20	
Moderate and severe	2.67	^a^P = 0.5426	2.81	^a^P = 0.3496	2.40	

Number and event rate (per patient year) of moderate and severe exacerbations in each treatment group. ^a^P values shown are from comparison of event rate with the placebo group

### Secondary outcomes

#### COPD exacerbations over the 12-week treatment period, and the first and last 24-week post-treatment periods

The exacerbation rate appeared slightly lower in the two active treatment arms compared with the placebo group during the active treatment period, however, this difference was not statistically significant (1.64 per patient year in the roxithromycin/doxycycline group and 1.74 per patient year in the roxithromycin only group versus 2.25 per patient year in the placebo group, p = 0.1709 and p = 0.2545 respectively) (Table 3). There were no statistically significant differences between either of the two active treatments groups and the placebo group in the annualized moderate and severe event rates over either 24-week post-treatment period. When the analysis was corrected for factors significantly associated with exacerbation rate (age, weight, smoking status, FEV_1_ percentage predicted and the use of concomitant antibiotics), there remained no statistically significant difference among the groups.

### FEV_1_ and Forced Vital Capacity (FVC) over 60-week period

**Fig. 2:**
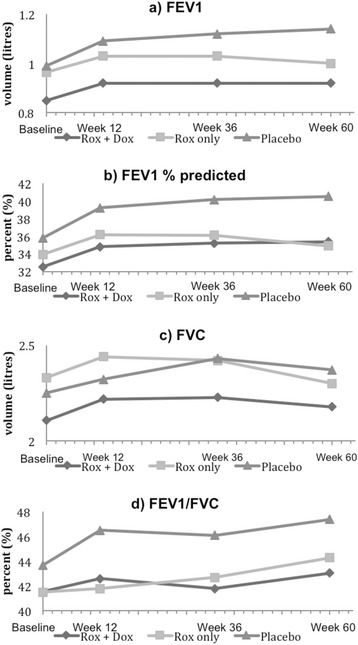
Time-course of spirometry by treatment groups. Trend from baseline to week 12 (end of treatment), week 36 and week 60 (end of follow up period) for **a**) mean absolute FEV_1_, **b**) FEV_1_ percentage predicted, **c**) mean absolute FVC and **d**) FEV_1_/FVC, by treatment groups

By the end of the active treatment period (Fig. 2a), FEV_1_ increased by 5.5 % (47 mls) relative to baseline in the roxithromycin/doxycycline group, 5.9 % (57 mls) in the roxithromycin only group and 8.4 % (83 mls) in the placebo group. This remained more or less the same at 24 weeks and 48 weeks after treatment. These small improvements in FEV_1_ over the 60-week study period were statistically significant in all groups (p < 0.0001). Overall there was no significant difference among the treatment groups at the end of the study (p = 0.278). With regard to the change seen over time, there was no significant difference among the treatment groups (p = 0.539). By the end of the active treatment period (Fig. 2c), FVC increased by 2.8 % (60 mls) relative to baseline in the roxithromycin/doxycycline group, 3.9 % (90 mls) in the roxithromycin only group and 1.9 % (43 mls) in the placebo group but these were not statistically significant (p = 0.3441), and overall there was no significant difference among the treatment groups (p = 0.7192).

### Chronic Respiratory Disease Questionnaire (CRQ) scores over 60-week period

There were significant changes in the scores of all four CRQ domains relative to baseline in each treatment group over the 12-week active treatment and the 48-week post-treatment period (Additional file 1: Table S1). However there were no significant differences in any of the four domains among the treatment groups, or in the proportions of subjects meeting the minimum clinically important difference.

### Adverse events

Table S2 summarises the adverse events considered related to the study medication (Additional file 1: Table S2). The most common adverse event was nausea in both active treatment groups. There was only one case of abnormal electrocardiogram (ECG) deemed related to study medication in the roxithromycin/doxycycline group. Most events were mild and no deaths were related to the study medication.

## Discussion

The major finding of this study was the lack of effect on the frequency of COPD exacerbations following a 12-week treatment with roxithromycin either alone or in combination with doxycycline in the COPD population with serological evidence of previous *C. pneumoniae* infection when compared with placebo. In addition there was no significant difference among treatment groups with regard to any of the other efficacy parameters examined i.e. lung function and quality of life scores.

At the time that this study was undertaken there was a focus on the role of chronic infection with *C. pneumoniae* in the pathogenesis of chronic respiratory disorders such as COPD. Subsequently, interest in *C. pneumoniae* as an important cofactor in the aetiology of respiratory diseases has dwindled [8, 10]. Previous studies have shown that *C. pneumoniae* seropositivity is seen in up to 77 % of stable COPD patients and is not significantly related to FEV_1_ percentage predicted or exacerbation frequency [9, 11]. Although the included cohort with positive *C. pneumoniae* serology had a greater degree of airflow obstruction and more exacerbations than the excluded patients, these findings are unlikely to have any impact on the efficacy endpoints in this study or the interpretation of these. Therefore it is plausible that the results of this clinical trial might be generalised to other COPD patients with frequent exacerbations.

The Cochrane review by Staykova et al published in 2003 analysed nine randomised controlled trials (all undertaken prior to 1970) of prophylactic antibiotic therapy in subjects with chronic bronchitis [2]. Tetracycline antibiotics predominated the clinical trials (including one trial with tetracycline/macrolide combination) with a variable treatment duration averaging 5 months. The 8.6 % reduction of exacerbations per patient year with treatment was not statistically significant. There was no significant reduction in exacerbation rate seen with tetracycline only studies. The finding of the lack of effect of prophylactic antibiotic use seen in our study is therefore in agreement with the findings from these historical prophylactic antibiotic studies.

Since this study was undertaken, a number of prophylactic antibiotic trials have been undertaken using newer macrolides and fluoroquinolones [4, 5, 12–17]. These studies followed the documented efficacy of macrolide therapy in the cystic fibrosis (CF) population and more recently in non-CF bronchiectasis [18, 19]. Recent studies of azithromycin or erythromycin in non-CF bronchiectasis showed that 6 to 12 month treatment with these macrolides reduced the rate of exacerbations and prolonged the duration to first exacerbations significantly [20–22].

In the updated Cochrane review on prophylactic antibiotic therapy in COPD, the largest randomised controlled trial by Albert et al studied 12 months of once daily 250 mg azithromycin in 1577 COPD patients [3, 5]. In accordance with our findings, there was no statistically significant difference in COPD hospitalisations (0.34 vs. 0.49 per patient year for azithromycin and placebo respectively), although the overall exacerbation rate was lower in the active treatment group (1.48 vs. 1.83 per patient year) [5]. Unlike our study, half of the patients did not have history of frequent exacerbations, hence the lower annualised exacerbation rates and longer duration to first exacerbation. A more recent trial by Uzun et al used 500 mg azithromycin three times a week for 12 months in a smaller cohort of 92 COPD patients with history of three or more exacerbations in the previous year [4]. The overall exacerbation rate was significantly lower in the azithromycin group than placebo (1.94 vs. 3.22 per patient year) as was the duration to first exacerbation (130 vs. 59 days). However, the rates of severe exacerbation and hospitalisation were not statistically different. They did not find any significant improvement in lung function and overall health-related quality of life. Both of these one-year azithromycin trials showed that the significant decrease in exacerbation rates between the active and placebo groups was seen within 3 months of treatment [4, 5]. Although we observed a similar trend in our study, our results were not statistically significant.

A more recent randomised controlled trial by Brill et al of three different antibiotic regimens (including azithromycin) in patients with stable COPD found that there was no significant reduction in sputum bacterial load following 3 months of treatment [23]. This suggests that azithromycin’s action in reducing the risk of exacerbations in COPD patients may be due to its immunomodulatory effect as suggested by previous authors [24].

Even though the number of patients who did not wish to continue on the study medications was higher in the roxithromycin/doxycyline group than the roxithromycin and placebo groups, their decisions were not related to the adverse events. We found that the adverse events from prophylactic antibiotics were tolerable and apart from nausea, were comparable between the treatment groups. Macrolides, however, are known to have cardiac side effects (e.g. prolonged QT interval) and could potentially cause malignant arrhythmia, such as torsades des pointes. A retrospective study on azithromycin by Ray et al showed that its use was associated with increased cardiovascular deaths [25]. Erythromycin and azithromycin are also potent inducers of antimicrobial resistance in both non-CF bronchiectasis and COPD studies [5, 20, 21]. Herath et al, although unable to analyse the development of antibiotic resistance in their review, conveyed their concern on the impact of bacterial macrolide resistance as observed in the study by Albert et al. [3 5]. The more recent trials by Uzun et al and Brill et al have also shown the development of antimicrobial resistance [4, 23]. This resistance may limit treatment options for acute bacterial respiratory infection and even increase respiratory mortality. Our study in which antibiotics were given for twelve weeks did not examine antimicrobial resistance.

One of the strengths of our study is that it looked at both the 12-week on-treatment period and the 48-week run-out period, in order to see if any sustainable impact was present beyond the treatment period. Another is that even though the number of patients recruited did not reach the target, the dropout rate was lower than expected and therefore the number of patients was adequate in order to achieve statistical significance. This is one of the largest double-blind placebo-controlled trials undertaken on antibiotic prophylaxis in COPD in recent years.

This study has several limitations. There was a delay in getting the study prepared for publication. This was due a combination of factors which included the untimely death of the principal investigator. However, there was no delay in the analysis of the data and the results remained valid throughout this period. Although the study only included participants seropositive to *C. pneumonia,* as discussed above, our study cohort is likely to be representative of COPD patients with a history of frequent exacerbations. The duration of antibiotic therapy of twelve weeks is shorter than other studies [3–5], which could limit any possible antimicrobial and anti-inflammatory effects of these antibiotics.

## Conclusions

Twelve weeks of treatment with roxithromycin alone or in combination with doxycycline was tolerable but did not reduce the frequency of exacerbations in patients with COPD either during the treatment period or in the subsequent 48 weeks. The outcome of this study certainly does not justify the use of these antibiotics to prevent COPD exacerbations. There remains the need for a more targeted approach to reduce exacerbations in COPD patients with frequent exacerbations than the use of prophylactic antibiotics.

## Methods

This study was undertaken between February 2000 and April 2002 in 16 centres across Australia and New Zealand. It was designed specifically to look at the role of antibiotic therapy active against *Chlamydia pneumoniae* in a COPD population with serological evidence of previous *C. pneumoniae* infection. The study endpoints and the statistical analysis (apart from those in relation to *C. pneumoniae*), and the sample size calculation were as per the originally designed study protocol (Additional file 2).

### Study participants

The inclusion criteria were subjects aged 45 years or older, meeting spirometric criteria for COPD (FEV_1_ ≤ 70 % predicted, ratio of FEV_1_ over FVC (FEV_1_/FVC) ≤ 60 %, reversibility of ≤ 10 % of predicted FEV_1_ or ≤ 200 ml if predicted FEV_1_ ≤ 2 L); smoking history ≥ 20 pack years; and at least three confirmed moderate or severe COPD exacerbations in the past two years (i.e. requiring treatment with antibiotics and/or oral corticosteroids and/or hospitalisation). In view of the original hypothesis, an additional inclusion criterion was positive serology for *C. pneumoniae* (IgG antibody titre ≥1:64).

The exclusion criteria were pulmonary disease other than COPD; treatment with antibiotics, exacerbation or an investigational drug in the four weeks before randomisation; pregnancy (serum pregnancy test) or breast feeding; history of hypersensitivity to macrolides, tetracyclines, beta-lactams or sulfamethoxazole:trimethoprim; serious cardiovascular, hepatic, renal or other systemic diseases; known long QT syndrome or corrected QT interval (QTc) >450 ms, sick sinus syndrome, bradycardia (<50 beats per minute) or severe hypokalaemia; epilepsy; treatment with medicine known to have important interaction with macrolides or tetracyclines; impaired hepatic function (aspartate aminotransferase or alanine aminotransferase ≥ 2 times of the upper limit of normal (ULN), alkaline phosphatase ≥ 1.25 times the ULN, bilirubin >2 times the ULN and albumin <30 g/L); or unlikely to comply.

Written informed consent was obtained from each subject prior to enrolment in the study. Ethics approval was obtained from the local ethics committees of each of the participating sites. This trial was retrospectively registered with the Australian New Zealand Clinical Trials Registry (ANZCTRN12615000052538).

### Interventions

This randomised placebo-controlled double-blind double-dummy parallel group study compared three different treatments administered over a three month period: 1) the macrolide roxithromycin 300 mg alone; 2) the combination of roxithromycin 300 mg daily and the tetracycline antibiotic doxycycline 100 mg daily; or 3) matching placebo. Based on the original hypothesis, the combination of roxithromycin and doxycycline would be more effective in eradicating *C. pneumoniae* than roxithromycin alone and would lead to decreased exacerbation rates and improved lung function [26, 27].

Hoechst Marion Roussel and Douglas Pharmaceuticals manufactured roxithromycin and doxycycline tablets respectively, together with their identical placebo tablets. Study medication was packed by Hoechst Marion Roussel in bottles labelled with the randomisation and batch numbers. The investigators, pharmacists and subjects were blinded to the study medication in these bottles.

After a 2-week run-in period, each eligible patient was assigned a sequential subject number followed by randomisation number provided by Hoechst Marion Roussel, Australia. Subjects were supplied with one of the three treatments according to their randomisation number. After completing the initial 12-week antibiotic period, participants were followed up for a further 48 weeks, for a total of 60 weeks.

### Outcome variables

The primary outcome variable was the frequency of moderate and severe exacerbations (defined below) over the 48-week post treatment period. The secondary outcome variables were the number of exacerbations of COPD over the active treatment period, and the first and last 24-week periods after treatment; spirometric volume changes (FEV_1_ and FVC); Chronic Respiratory Disease Questionnaire (CRQ) scores, and adverse events over the 60-week period. Minimum clinically relevant changes for each domain of the CRQ scores were defined as a change of 3 points for dyspnoea, 4 points for emotional function, 2 points for fatigue and mastery (as per Guyatt) [28, 29].

An acute exacerbation was defined by either at least 2 out of 3 of the following, on 3 consecutive days or more, change in sputum production; change in sputum purulence; and change in breathlessness; or diagnosed and treated by the investigator based on clinical symptoms. Exacerbation severity was defined as ‘mild’ if it was self-managed by the patient at home (e.g. increase in bronchodilator and/or non-prescription medication use); ‘moderate’ if it required treatment with antibiotics and/or an increase in dose of, or initiation of corticosteroids by a medical practitioner; or ‘severe’ if it resulted in hospitalisation or death due to an exacerbation of COPD.

### Data collection

A full medical history was obtained at the screening visit. A full physical examination was performed at randomisation and at the end of treatment. CRQ and spirometry (performed according to the European Respiratory Society guidelines) were done at week 0, 12, 36 and 60. A 12-lead ECG was performed on all patients at randomisation, during treatment and at the end of treatment. QTc was calculated using Bazett’s formula. Any adverse events observed by the investigator or reported by the subject were documented. Details of any exacerbations, changes in medication, and adverse events were collected from the Daily Diary Cards provided to each subject and from regular telephone assessments. All subjects were advised to contact site investigators if they were experiencing any exacerbations for details of symptoms, and information on doctor visits or hospitalisations. Sputum microbiology and antimicrobial resistance were not incorporated into this study.

### Statistical analysis

Comparison of baseline characteristics was done using the chi-squared test, student’s t-test or Wilcoxon rank-sum test. Comparison of event rates (number of events/exposure patient days) among the three treatment arms was done using a Poisson model. Analysis of variance models were used to test for differences in numbers of exacerbations among the three treatment groups. Statistical analyses for all secondary outcome variables were performed at the end of the 60-week study period in comparison with baseline values. Analysis of time to first moderate or severe exacerbations was performed using log-logistic distribution model. CRQ scores were analysed using autoregressive models and likelihood-ratio tests. All randomised patients were included in the intention-to-treat analyses. The subjects who had withdrawn from the study medication remained for the full assessment period and were reviewed at the final visit day, as far as possible. Data were entered and managed in a Clintrial database. The SAS statistical package was used for all analyses. There was no delay in the analyses of data for the study endpoints and they were performed right after completion of the study.

### Sample size justification

The mean number of moderate and severe exacerbations per year was estimated to be 2.5 (SD 1) in each group. An analysis of variance-based F-test would require 83 subjects in each treatment group to have 90 % power to detect a difference in the exacerbation rate of 0.5 per year at the 5 % significance level. Thus a total of 249 subjects would be required. If a dropout rate were approximately 20 %, 312 subjects would be required.

## Additional files

Additional file 1: Table S1. Chronic Respiratory Questionnaire (CRQ) scores by treatment groups, and **Table S2** Adverse events by treatment groups. (DOCX 85 kb) Additional file 2:
**Study Protocol.** (PDF 2741 kb)

## Abbreviations

CF: Cystic fibrosis
CI: Confidence intervals
COPD: Chronic obstructive pulmonary disease
CRQ: Chronic Respiratory Disease Questionnaire
ECG: Electrocardiogram
FEV_1_: Forced expiratory volume in 1 second
FVC: Forced vital capacity
FEV_1_/FVC: Ratio of forced expiratory volume in 1 second over forced vital capacity
OR: Odds ratio
RR: Relative risk
SD: Standard deviation
ULN: Upper limit of normal
