# Supplementary material for: A double-blind, randomised, placebo-controlled study of roxithromycin and doxycycline combination, roxithromycin alone, or matching placebo for 12 weeks in adults with frequent exacerbations of chronic obstructive pulmonary disease
Source: J Negat Results Biomed. 2015 Sep 7;14:15. doi: 10.1186/s12952-015-0034-8 (PMC4562194; doi:10.1186/s12952-015-0034-8)
Supplement: Additional file 1: Table S1. — Chronic Respiratory Questionnaire (CRQ) scores by treatment groups, and Table S2 Adverse events by treatment groups. (DOCX 85 kb) [file 12952_2015_34_MOESM1_ESM.docx]

## Supplement Table 1 - Chronic Respiratory Questionnaire (CRQ) scores by treatment groups.

Chronic Respiratory Questionnaire (CRQ) scores over the 60-week period by treatment groups including visit effect, treatment effect, visit interaction and proportion of patients reaching minimum clinically relevant change in each domain at the end of the study (Week 60).

| **Domain** | **Treatment** | **Baseline**  **Mean, SD, n** | **Week 12**  **Mean, SD, n** | **Week 36**  **Mean, SD, n** | **Week 60**  **Mean, SD, n** | **Visit effect**  **(p value)** | **Treatment effect (p value)** | **Visit interaction (p value)** | **Proportion of patients reaching minimum clinically relevant change (p value)** | |
| --- | --- | --- | --- | --- | --- | --- | --- | --- | --- | --- |
| **Dyspnoea** | Roxithromycin + Doxycycline | 15.45 (4.8, 101) | 17.7 (6.3, 93) | 17.2 (7.2, 82) | 17.2 (7.3, 78) | <0.0001 | 0.1321 | 0.4598 | 30% | 0.0561 |
|  | Roxithromycin only | 15.7 (4.8, 97) | 17.4 (6, 94) | 16.7 (5.3, 90) | 15.5 (5.4, 87) |  |  |  | 26% |  |
|  | Placebo | 16.7 (5.2, 93) | 18.9 (6.8, 88) | 18.3 (6.9, 83) | 18.7 (7.8, 80) |  |  |  | 39% |  |
| **Fatigue** | Roxithromycin + Doxycycline | 14.8 (4.9, 101) | 15.3 (5.45, 93) | 15.3 (5.8, 82) | 15.7 (5.9, 78) | 0.0011 | 0.4266 | 0.4501 | 32% | 0.3961 |
|  | Roxithromycin only | 15.5 (4.5, 97) | 16.2 (5.4, 94) | 15.6 (5.3, 90) | 15 (5.6, 87) |  |  |  | 30% |  |
|  | Placebo | 14.6 (4.8, 93) | 15.7 (5.7, 89) | 15.3 (5.1, 84) | 15.7 (5.7, 84) |  |  |  | 38% |  |
| **Emotional Function** | Roxithromycin + Doxycycline | 33 (8.1, 101) | 23.3 (6.15, 93) | 22.8 (6.7, 82) | 22.9 (6.7, 78) | <0.0001 | 0.3027 | 0.4419 | 22% | 0.8255 |
|  | Roxithromycin only | 33.1 (8.3, 97) | 24.6 (6.7, 94) | 23.7 (6.9, 90) | 23.8 (6.8, 87) |  |  |  | 20% |  |
|  | Placebo | 32.2 (8.6, 93) | 24.4 (6.4, 88) | 23.6 (6.4, 83) | 23.8 (6.4, 81) |  |  |  | 23% |  |
| **Mastery** | Roxithromycin + Doxycycline | 19.3 (5.6, 101) | 19.8 (5.7, 93) | 20.1 (5.4, 82) | 19.4 (5.8, 78) | <0.0001 | 0.1688 | 0.1522 | 35% | 0.1107 |
|  | Roxithromycin only | 19.4 (5.3, 97) | 20.8 (5.3, 94) | 20.4 (5.6, 90) | 19.5 (6, 87) |  |  |  | 27% |  |
|  | Placebo | 19.4 (5.5, 93) | 20.6 (5.6, 89) | 20.7 ( 5.2, 84) | 21.3 (5.1, 81) |  |  |  | 38% |  |

## Supplement Table 2 - Adverse events by treatment groups.

Adverse events considered related to the study medication.

|  | **Roxithromycin/**  **Doxycycline** | | **Roxithromycin alone** | | **Placebo** | | **p-value** |
| --- | --- | --- | --- | --- | --- | --- | --- |
| Number (%) of patients with suspected related event(s) | 31 | 30.69% | 33 | 34.02% | 13 | 13.83% | 0.012 |
| Total number of suspected related events | 44 | 38.94% | 51 | 45.13% | 18 | 15.93% | 0.001 |
| **Intensity, number of events (%):** |  |  |  |  |  |  |  |
| Mild | 33 | 75.00% | 22 | 43.14% | 14 | 77.78% | 0.003 |
| Moderate | 9 | 20.45% | 20 | 39.22% | 3 | 16.67% | 0.004 |
| Severe | 2 | 4.55% | 9 | 17.65% | 1 | 5.56% | 0.004 |
| **Adverse events, number of events (%):** |  |  |  |  |  |  |  |
| Nausea | 12 | 27.27% | 13 | 25.49% | 1 | 5.56% | 0.001 |
| Diarrhoea | 2 | 4.55% | 3 | 5.88% | 1 | 5.56% | 0.943 |
| Headache | 4 | 9.09% | 1 | 1.96% | 1 | 5.56% | 0.113 |
| Abdominal pain | 3 | 6.82% | 1 | 1.96% | 1 | 5.56% | 0.247 |
| Reflux | 2 | 4.55% | 1 | 1.96% | 0 | 0.00% | 0.066 |
| Vomiting | 1 | 2.27% | 3 | 5.88% | 0 | 0.00% | 0.03 |
| Abnormal liver function | 1 | 2.27% | 2 | 3.92% | 0 | 0.00% | 0.135 |
| Abnormal ECG | 1 | 2.27% | 0 | 0.00% | 0 | 0.00% | 0.135 |
| Rash | 1 | 2.27% | 1 | 1.96% | 1 | 5.56% | 0.202 |
| Dyspnoea | 0 | 0.00% | 1 | 1.96% | 2 | 11.11% | <0.001 |
| Dizziness | 0 | 0.00% | 4 | 7.84% | 0 | 0.00% | <0.001 |
| Oral candidiasis | 0 | 0.00% | 2 | 3.92% | 3 | 16.67% | <0.001 |
| GI upset | 0 | 0.00% | 2 | 3.92% | 2 | 11.11% | 0.002 |
