# Supplementary material for: A double-blind, randomised, placebo-controlled study of roxithromycin and doxycycline combination, roxithromycin alone, or matching placebo for 12 weeks in adults with frequent exacerbations of chronic obstructive pulmonary disease
Source: J Negat Results Biomed. 2015 Sep 7;14:15. doi: 10.1186/s12952-015-0034-8 (PMC4562194; doi:10.1186/s12952-015-0034-8)
Supplement: Additional file 2: — Study Protocol. (PDF 2741 kb) [file 12952_2015_34_MOESM2_ESM.pdf]

Hoechst Marion Roussel

CLINICAL STUDY PROTOCOL

A DOUBLE-BLIND, RANDOMISED, PLACEBO-CONTROLLED STUDY OF THE CLINICAL EFFICACY AND TOLERABILITY OF ROXITHROMYCIN ALONE AND IN COMBINATION WITH DOXYCYCLINE FOR 12 WEEKS IN ADULTS WITH CHRONIC OBSTRUCTIVE PULMONARY DISEASE (COPD) AND SEROLOGICALLY PROVEN INFECTION WITH *CHLAMYDIA PNEUMONIAE.*

RU28965/4024

Multi-national Study

Local Safety Officer (AUS/NZ)
Brett Handley
Hoechst Marion Roussel
27 Sirius Road
LANE COVE NSW 2066
AUSTRALIA
Tel: +61 2 9422 6444
Fax: +61 2 9422 6441

Sponsor:
Hoechst Marion Roussel Australia Pty Ltd
27 Sirius Road
LANE COVE NSW 2066
AUSTRALIA

Study Manager
Louise Hayman
Hoechst Marion Roussel
27 Sirius Road
LANE COVE NSW 2066
AUSTRALIA
Tel: +61 2 9422 6477
Fax: +61 2 9422 6441
Mobile: +61 412 252 758

Site Monitor
(Australia)
Rebecca Gibson
Hoechst Marion Roussel
27 Sirius Road
LANE COVE NSW 2066
AUSTRALIA
Tel: +61 2 9422 6303
Fax: +61 2 9422 6441
Mobile: +61 411 439 754

Site Monitor
(New Zealand)
Julie Siegert
Hoechst Marion Roussel
101 Station Rd
Penrose
NEW ZEALAND
Tel: +64 9 526 0376
Fax: +64 9 526 1777
Mobile: +64 21 829 889

This protocol is the confidential information of Hoechst Marion Roussel and is intended solely for the guidance of the clinical investigation. This protocol may not be disclosed to parties not associated with the clinical investigation or used for any purpose without the prior written consent of Hoechst Marion Roussel.

CONFIDENTIAL

Total pages in document: 61

TABLE OF CONTENTS

TABLE OF CONTENTS 2

PROTOCOL OUTLINE 5

STUDY SCHEDULE 8

ABBREVIATIONS AND DEFINITIONS 9

1 INTRODUCTION AND STUDY RATIONALE 10

2 STUDY OBJECTIVES 14
- 2.1 **Primary objective** 14
- 2.2 **Secondary objectives** 14

3 STUDY DESIGN 15

4 SELECTION OF SUBJECTS 16
- 4.1 **Number of subjects** 16
- 4.2 **Inclusion criteria** 16
- 4.3 **Exclusion criteria** 17

5 STUDY TREATMENTS 19
- 5.1 **Details of study treatments** 19
- 5.2 **Dosage schedule** 19
- 5.3 **Treatment assignment** 19
- 5.4 **Blinding, packaging, and labelling** 20
- 5.5 **Supplies and accountability** 21
- 5.6 **Compliance** 21

6 PRIOR AND CONCOMITANT ILLNESSES AND TREATMENTS 22
- 6.1 **Prior and concomitant illnesses** 22
- 6.2 **Prior and concomitant treatments** 22

7 STUDY PROCEDURES AND SCHEDULE 24
- 7.1 **Overview of data collection** 24
- 7.2 **Description of study days** 25
  - 7.2.1 Prestudy screening 25
  - 7.2.2 Study days 26
  - 7.2.3 Liaison throughout Study Period 30
- 7.3 **Methods of data collection** 30
  - 7.3.1 Efficacy data 30
  - 7.3.2 Safety data 31
  - 7.3.3 Pharmacokinetic/Pharmacodynamic data 32
  - 7.3.4 Quality-of-life 32
  - 7.3.5 Pharmacoeconomic data 32

8 ADVERSE EVENTS 34
8.1 **Definitions** 34
8.1.1 Adverse event 34
8.1.2 Serious adverse events 34
8.1.3 Alert terms 35
8.2 **Period of observation** 35
8.3 **Documentation and reporting of adverse events by investigator** 35
8.4 **Immediate reporting by investigator to sponsor** 36

9 WITHDRAWALS 37
9.1 **Withdrawal of subjects** 37
9.2 **Replacement of subjects** 37

10 EMERGENCY PROCEDURES 38
10.1 **Emergency sponsor contact** 38
10.2 **Emergency identification of study medication** 38
10.3 **Emergency treatment** 38

11 STATISTICAL PROCEDURES 39
11.1 **Analysis variables** 39
11.1.1 Primary outcome variables 39
11.1.2 Secondary outcome variables 39
11.2 **Study populations** 40
11.3 **Statistical methods** 40
11.4 **Interim analysis** 40
11.5 **Sample size justification** 40

12 ETHICAL AND LEGAL ASPECTS 41
12.1 **Good clinical practice** 41
12.2 **Delegation of investigator responsibilities** 41
12.3 **Subject information and informed consent** 41
12.4 **Confidentiality** 42
12.5 **Protocol amendments** 42
12.6 **Approval of the study protocol and amendments** 42
12.7 **Ongoing information for independent ethics committee/ institutional review board** 42
12.8 **Premature closure of the study** 43
12.9 **Record retention** 43
12.10 **Liability and insurance** 43

13 STUDY MONITORING AND AUDITING 44
13.1 **Study monitoring** 44
13.2 **Source data verification and on-site audits** 44

14 DOCUMENTATION AND USE OF STUDY FINDINGS 45
14.1 **Documentation of study findings** 45
14.2 **Use of study findings** 45

15 STUDY DURATION AND DATES 47

16 DECLARATIONS OF SPONSOR AND INVESTIGATOR 48
16.1 Declaration of sponsor 48
16.2 Declaration of investigator 49

17 REFERENCES 50

APPENDIX A 52
Cockcroft Gault Formula

APPENDIX B 53
Daily Symptom Score - Exacerbations

APPENDIX C 54
Regimen For Administration Of Medicine – Guidelines

APPENDIX D 55
QUALITY OF LIFE – The Chronic Respiratory Disease Index Questionnaire

APPENDIX E 56
Spirometry

APPENDIX F 57
Diagnosis of *Chlamydia pneumonia* Infection

APPENDIX G 58
Sputum Collection

APPENDIX H 59
Laboratory Data

APPENDIX I 60
Specimen Collection and Storage

APPENDIX J 61
Microbiological evaluation of sputum

PROTOCOL OUTLINE

RU 29865A/4024

**Title**	A double-blind, randomised, placebo-controlled study of the clinical efficacy and tolerance of roxithromycin 300 mg daily alone and in combination with doxycycline 100 mg daily for 12 weeks in adults with Chronic Obstructive Pulmonary Disease (COPD) and serologically proven infection with *Chlamydia pneumoniae.*
**Investigator(s), Study site(s)**	Multicentre study (in Australia and New Zealand)
**Phase**	III
**Indication**	Adults with symptomatic COPD and positive serology for *Chlamydia pneumoniae.*
**Objectives**	Clinical efficacy and safety of roxithromycin 300 mg daily for 12 weeks and of the combination of roxithromycin 300 mg daily and doxycycline 100 mg daily for 12 weeks in the reduction of moderate or severe COPD exacerbations in the 48 weeks following treatment.
**Design**	Placebo-controlled, double-blind, randomised, parallel group study. Screening visit, 12 weeks active or placebo treatment followed by 48 weeks follow-up.

Population Inclusion Criteria

- Age ≥ 45 years.
- COPD with $FEV_1 \leq 70\%$ of predicted, $FEV_1/FVC \leq 60\%$ and reversibility less than 15% and/or 200 mL.
- Smoking history of ≥ 20 pack years.
- At least three confirmed COPD exacerbations in the past two years
- Positive serology for *Chlamydia pneumoniae* (IgG antibody titre ≥1:64)
- Informed consent

Exclusion Criteria

- Pulmonary disease other than COPD
- Treatment with antibiotics in the 4 weeks before randomisation (Visit 2).
- Exacerbation in the 4 weeks before randomisation (Visit 2)
- Pregnancy (serum pregnancy test) or breast feeding
- Hypersensitivity to macrolides, tetracyclines, beta-lactams or sulfamethoxazole; trimethoprim
- Serious cardiovascular, hepatic, renal or other systemic diseases
- Patients with a known long QT syndrome or QTc >450 ms, sick sinus syndrome, bradycardia (<50 beats per minute) or severe hypokalaemia
- Patients diagnosed with epilepsy
- Treatment with an investigational drug in the 4 weeks before randomisation (Visit 2)
- Treatment with medicine known to have important interaction with macrolides or tetracyclines
- Unreliable or unlikely to comply

Sample size

83 subjects to complete in each group. Assuming a drop out rate of 20% then 312 subjects will be recruited (104 per group).

Treatments

Roxithromycin 300 mg daily, the combination of roxithromycin 300 mg daily and doxycycline 100 mg daily and matching placebos.

Efficacy data

Primary

- Reduction in frequency and severity of acute infective exacerbations of COPD.

Secondary

- Improvement in Quality of Life Score (CRQ)
- Improvement in FEV_1 and FVC
- Titres of IgG and IgA antibodies for *C. pneumoniae*

- Eradication of *C. pneumoniae* from monocytes as determined by PCR
- Eradication of *C. pneumoniae* from sputum as determined by PCR
- Reduction in secretory IgA to *C. pneumoniae* in sputum
- Effect on sputum microbiology (selected centres)

Safety data

Clinical

- Recording of adverse events, spontaneous and elicited.

Laboratory

- Haematology, biochemistry and hepatic function.

Health resource use data

Outcome measures

- Number of hospitalisations due to COPD
- Number of visits to medical practitioners and other health professionals due to COPD
- Alterations to drug usage

Statistical procedures

Statistical analysis for all primary and secondary efficacy parameters will be performed at 24 and 48 weeks after treatment in comparison with baseline. Analysis of variance models will be pursued to test for differences in numbers of exacerbations between the 3 treatment groups.

Study duration and dates

End of 1999 to late 2001

STUDY SCHEDULE

Study Week	-2 to -4	0	2	4	6	9	12	16	20	24	28	32	36	40	44	48	52	56	60
**Weeks Post Treatment**								4	8	12	16	20	24	28	32	36	40	44	48
**Visit[5]**	V1	V2[1]	V3		V4		V5			V6			V7			V8			V9
**Telephone Call**				T		T		T	T		T	T		T	T		T	T	
**Eligibility (incl./excl.)**	X	X																	
**Informed Consent**	X																		
**Demographics**	X																		
**Medical History**	X																		
**Spirometry**	X	X					X						X						X
**Reversibility**	X																		
**Physical Examination**		X					X												
**12 Lead ECG**		X	X		X[3]		X			X[4]									
**Pregnancy Test**	X																		
**Serology**	X						X						X						X
**Safety Bloods**	X		X	X	X	X	X	X											
**Sputum for sIgA & PCR[2]**	X						X						X						X
**Monocyte PCR**	X						X						X						X
**QoL (CRQ)**		X					X						X						X
**Conc. Medications**		X	X	X	X	X	X	X	X	X	X	X	X	X	X	X	X	X	X
**Adverse Events**		X	X	X	X	X	X	X	X	X	X	X	X	X	X	X	X	X	X
**Exacerbations of COPD**		X	X	X	X	X	X	X	X	X	X	X	X	X	X	X	X	X	X
**Diary Card (provide)**	X	X	X		X		X			X			X			X			
**Issue ID Patient Card**		X																	
**Dispense Study Meds.**		X	X		X														
**Compliance Check**			X		X		X												

1Visit 2 (randomisation) should occur within 2-4 weeks of visit 1
2Spontaneous sputum will be obtained, however, the investigator may induce sputum if necessary.
3If ECG is abnormal at visit 3 (on therapy), this should be repeated at visit 4, otherwise the next ECG will be done at visit 5
4If ECG is not done or is abnormal at end of therapy visit (visit 5), a further ECG should be performed at visit 6
5Visits 3, 4 & 5 should occur ± 3 days, all other visits should occur ± 5 days
6Spirometry only required at Visit 2 if not completed at Visit 1

ABBREVIATIONS AND DEFINITIONS

ALT	Alanine aminotransferase
AST	Aspartate aminotransferase
COPD	Chronic Obstructive Pulmonary Disease
CRQ	Chronic Respiratory Disease Questionnaire
ERSCS	European Respiratory Society Consensus Statement
FEV_1	Forced Expiratory Volume in One Second
MIF	Microimmunofluorescence
OTC	Over The Counter therapy
PCR	Polymerase Chain Reaction
QOL	Quality of Life
RTI	Respiratory Tract Infection
FVC	Forced Vital Capacity

1 INTRODUCTION AND STUDY RATIONALE

Chronic Obstructive Pulmonary Disease (COPD) is a common condition with 15-20% of smokers going on to develop disabling breathlessness from COPD. In addition exacerbations of COPD are a common cause of hospitalisation. In the US in 1986 more than 13% of hospitalisations or almost 2 million hospitalisations resulted from COPD[1]. In New Zealand it is the second most common reason for admission to a medical ward. Clearly strategies to reduce the severity or frequency of exacerbations would be welcome. There is increasing evidence of an association between chronic infection with *Chlamydia pneumoniae* and COPD. These observations provide reason to believe that eradication of infection with *C. pneumoniae* might reduce the frequency of acute exacerbations of COPD.

A number of studies have looked for evidence of infection with *C. pneumoniae* in COPD. Beaty et al compared 44 patients admitted to hospital with acute exacerbations of COPD, 65 stable clinic patients with COPD and a control group of 24 subjects without COPD who were recruited from a urology clinic[2]. IgG antibodies to *C. pneumoniae* were found in 81% of patients admitted with acute exacerbations of COPD, 77% of the subjects with stable COPD and in 73% of the control subjects. The differences between the groups were not statistically significant but the authors did note that the prevalence of antibodies to *C. pneumoniae* in age and sex matched subjects (who did not have COPD) in other studies conducted in the same city was lower at 57%. Blasi et al compared 142 outpatients with exacerbations of COPD and 114 healthy controls[3]. They found that the prevalence of IgG antibodies to *C. pneumoniae* was 63% in the subjects with COPD and 46% in the control subjects. The difference was statistically significant (p=0.007) and the geometric mean titre of antibody was also higher in the patients with COPD.

The presence of IgG antibodies to *C. pneumoniae* does not, however, distinguish between previous infections and chronic infection. Studies on patients with atherosclerosis have provided clear evidence of persistent infection with *C. pneumoniae* in blood vessels. *C. pneumoniae* has been demonstrated in atherosclerotic plaques using immunohistochemistry and PCR[4,5]. In contrast it is very unusual to find *C. pneumoniae* in normal arteries. More recently *C. pneumoniae* has been cultured from the coronary artery of a patient undergoing heart transplantation[6] and from a carotid endarterectomy specimen[7]. It would not be surprising if chronic infection also occurred in the lung.

Evidence for chronic infection with *C. pneumoniae* in subjects with COPD comes from a study by von Hertzen et al[8]. They studied 40 subjects who had been hospitalised with an exacerbation of COPD and 14 subjects with COPD who had attended an outpatient clinic. 41 of the 54 subjects were classified as severe COPD ($FEV_1 < 50\%$) with the remaining 13 subjects ($FEV_1 > 50\%$) being classified as having mild to moderate disease. 23 patients who had been hospitalised for pneumonia were used as a control group. Tests on the sputum included secretory IgA (sIgA) antibodies to *C. pneumoniae* and PCR for *C. pneumoniae*. IgA antibody has a short half-life so its presence is likely to represent either recent infection or more probably chronic infection. PCR was positive in 59% of subjects with severe COPD, 40% of the subjects with mild-moderate COPD and 21.4% of the control group with pneumonia. sIgA was present in 80% of the group with severe COPD, 58.3% of those with mild-moderate COPD and 13.6% of the control group.

These findings would be consistent with the majority of subjects with COPD having chronic infection in the airways with *C. pneumoniae.*

Black et al (unpublished) have also addressed this question. Immunohistochemistry was performed on lung tissue obtained from subjects who had a lobe resected for bronchial carcinoma. Archival, formalin fixed, paraffin embedded tissue, which was obtained from a site remote from the carcinoma, was used. Subjects were classified as COPD ($FEV_1 < 80\%$ of predicted) or controls ($FEV_1 > 80\%$ of predicted) with 20 subjects in each group. In both groups 95% of the subjects had positive staining for *C.*

pneumoniae but the immunostaining was stronger in the COPD group. The control group had 8.7 positive cells per high power field (x40) compared with 12.8 cells per high power field in the COPD subjects ($p = 0.03$).

Macrophages are thought to have a key role in COPD so double staining for *C. pneumoniae* and macrophages was performed in the tissues described in the previous paragraph. In the control group 53% of the macrophages had double staining i.e. they also stained for *C. pneumoniae,* compared with 26% in the control group ($p=0.0008$).

The observation that alveolar macrophages are more likely to be infected with *C. pneumoniae* in COPD may be relevant. It has previously been shown that infection of monocytes by *C. pneumoniae* can induce the formation of cytokines such as Il-1 and TNF-α[9]. Increased levels of TNF-α are found in the sputum of subjects with COPD[10].

The evidence that chronic infection with *C. pneumoniae* may contribute to exacerbations of COPD comes from studies of prophylactic antibiotics in COPD. The largest such study was conducted in the United Kingdom by the Medical Research Council[11]. They recruited 497 subjects between 40 and 59 years with chronic bronchitis and an $FEV_1 > 1.4$ litres. (One of the criticisms of this study is that the subjects had mild COPD and this may have reduced the chance of seeing benefit with treatment.) Subjects were allocated to oxytetracycline 0.5 g or placebo once daily during the winter months (mid September - mid April) for 5 years. 373 subjects completed 5 years of treatment. During the study subjects were permitted to take one week's additional medication for exacerbations. Half the subjects were given chloramphenicol 0.5 g qid to take during an exacerbation. The other subjects were given sulphamethoxypyridazine 0.125 g qid (a sulphonamide) for exacerbations. Subjects were blinded as to whether they received chloramphenicol or sulphonamide for exacerbations.

There were in effect 4 treatment groups:

I Oxytetracycline with chloramphenicol for exacerbations

II Oxytetracycline with sulphonamide for exacerbations

III Placebo with chloramphenicol for exacerbations

IV Placebo with sulphonamide for exacerbations

There was a significant reduction in the number of exacerbations in Group I (oxytetracycline with chloramphenicol) but not in Group II. Over 5 years the exacerbation rate per subject was 2.69 for Group I, compared with 3.67 for Group II, 3.40 for Group III and 3.36 for Group IV.

In addition significantly fewer subjects in Group I (12) experienced purulent sputum compared with the other groups (28, 23 and 26). Because they were unable to provide an explanation their findings, the authors speculated that the difference between Group I and the other groups represented a Type I error. There is however a plausible therapeutic explanation for these observations.

Both tetracycline and chloramphenicol are active against *C. pneumoniae* whereas sulphonamides are not. Infections with *C. pneumoniae* can be difficult to eradicate. There are reports of individuals who have remained culture positive even after they were treated for their *C. pneumoniae* infection with several weeks of tetracycline or doxycycline[12]. It is entirely plausible that the combination of chloramphenicol and oxytetracycline would be more effective in suppressing or eradicating *C. pneumoniae* than either treatment alone. If chronic infection with *C. pneumoniae* increases the likelihood of an exacerbation then treatment with this combination would be more effective than either medicine alone at reducing the frequency of exacerbations. If this study had included subjects who experienced more frequent

exacerbations and if the combination of oxytetracycline and chloramphenicol had been administered for more than one week at a time, the benefits of treatment may have been even greater.

Treatment with chloramphenicol can lead to the development of aplastic anaemia and it would no longer be acceptable to use chloramphenicol in the treatment of patients with COPD. In addition to chloramphenical and tetracyclines the other groups of antibiotics with activity against *C. pneumoniae* are quinolones[13,14] and macrolides.

Infection of human bronchial epithelial cells with *C. pneumoniae* is associated with increased expression of mRNA for the pro-inflammatory cytokines IL-1, IL-8, IL-16 and RANTES. Similarly infection of human monocytes with *C. pneumoniae* leads to increased formation of the cytokines IL-1, IL-6 and TNF-α. If infection with *C. pneumoniae* can be eradicated it may in turn lead to a reduction in the inflammation which occurs in COPD. One would anticipate that a decrease in inflammation in the large airways would result in a diminution of the symptoms of chronic bronchitis and a reduction in exacerbations of chronic bronchitis. If inflammation in the small airways and the alveoli is decreased one may well see an effect on the decline in the lung function which occurs in COPD.

In all sites (large and small airways and alveoli) the predominant inflammatory cells are macrophages and CD8 lymphocytes. In addition in the large airways, neutrophils are found in the epithelium. The inflammation in the large airways is assumed to account for the cough and sputum which is seen in chronic bronchitis. Airflow obstruction in COPD is due to narrowing and obliteration of small airways and to the loss of elastic recoil, which results from the destruction of the alveolar walls (emphysema). It is probable that inflammation in the small airways and alveoli contributes to these changes.

Clearly there is a need for indirect methods of assessing inflammation in COPD. Sputum can be used to assess inflammation in the large airways. One of the limitations of sputum is that it cannot be used to assess inflammation in the small airways or the alveoli. Measurement of markers of inflammation in the peripheral blood is probably the best strategy for measuring inflammation in the lung periphery (short of lung biopsy). A proportion of subjects have increased levels of CRP, IL-8 and soluble receptors for TNF-α in venous blood. Measurement of these mediators could be used to monitor inflammation in COPD.

Superoxide formation by polymorphonuclear leukocytes is also increased in subjects with COPD, but from a practical point of view this is more difficult to measure because the granulocytes need to be isolated and then stimulated in vivo.

Neopterin is a marker of macrophage activation. Although there are no studies of neopterin in COPD, neopterin is elevated in pulmonary diseases associated with macrophage activation such as sarcoidosis.. It seems reasonable to postulate that serum and urinary neopterin may be elevated in subjects whose macrophages are infected with *C. pneumoniae.*

This study tests the hypothesis that chronic infection with *C. pneumoniae* predisposes patients to exacerbations of COPD. The effects of 12 weeks treatment with roxithromycin alone or in combination with doxycycline will be compared with placebo in a randomised, double-blind trial of patients with COPD and evidence of chronic infection with *C. pneumoniae.* The primary endpoint is the number of acute exacerbations of COPD over the ensuing year.

Further details can be found in the Product Information Sheets for roxithromycin and doxycycline.

Microbiological Evaluation

A microbiological study of the sputum will be undertaken to describe microbial patterns and observe any etiological shifts that occur after 12 weeks of antibiotic therapy. Sputum samples will be assessed for potentially pathogenic microorganisms. These would normally include *Haemophilus influenzae, Streptococcus pneumoniae* and *Moraxella catarrhalis*.[15, 16]

Studies with amoxycillin have shown that treatment can lead to colonisation of the oropharynx with resistant gram negative rods, with subsequent superinfections developing after treatment.[17,18] While there has been no evidence of this with macrolide antibiotics, this aspect of therapy will be evaluated. Results will provide an indication of the ability of roxithromycin either alone or in combination with levofloxacin to cause an etiological change in the flora colonising the respiratory tract.

2 STUDY OBJECTIVES

2.1 Primary objective

To evaluate the effect of 12 weeks' treatment with roxithromycin 300 mg daily alone or in combination with doxycycline 100 mg daily on the frequency of moderate and severe acute exacerbations, and the severity of all exacerbations, in subjects with Chronic Obstructive Pulmonary Disease in the 48 weeks following treatment.

2.2 Secondary objectives

To assess the effect of 12 weeks' treatment with roxithromycin 300 mg daily alone or in combination with doxycycline 100 mg daily in subjects with Chronic Obstructive Pulmonary Disease on:

- Frequency of all exacerbations (any severity)
- Scores on the Chronic Respiratory Disease Questionnaire (CRQ).
- FEV_1 and FVC.
- Titres of IgG and IgA antibodies for *C. pneumoniae.*
- Eradication of *C. pneumoniae* from the sputum as determined by PCR
- Reduction in secretory IgA to *C. pneumoniae* in sputum
- Eradication of *C. pneumoniae* from circulating monocytes as determined by PCR
- Sputum microbiology - to determine the effect of long term broad spectrum antibiotic therapy on the resident flora of sputum.

To assess the safety of 12 weeks' treatment with roxithromycin 300 mg daily alone or in combination with doxycycline 100 mg daily.

Economic outcomes (number of hospitalisations, number of visits to medical practitioners, alterations to drug usage and other health resource usage related to COPD) will be measured to evaluate the cost-effectiveness of treatments.

3 STUDY DESIGN

This is a multicentre, randomised, placebo-controlled, double-blind, double-dummy, parallel group study.

All patients screened who demonstrate positive serology and meet all inclusion / exclusion criteria will proceed to a 2 week run-in period, followed by a 12 week treatment period with a further 48 weeks of follow-up after the end of treatment.

Treatment **4 weekly follow-up by visits and telephone calls**

Visit #	V1	V2	V3	T	V4	T	V5	T	T	V6	T	T	V7	T	T	V8	T	T	V9
**Study Week**	-2	0	2	4	6	9	12	16	20	24	28	32	36	40	44	48	52	56	60

⇑ Screening
⇑ Randomisation

- ♦ Roxithromycin 300 mg OD / 12 weeks + Doxycycline 100 mg Placebo OD / 12 weeks, or
- ♦ Roxithromycin 300 mg OD / 12 weeks + Doxycycline 100 mg OD / 12 weeks, or
- ♦ Roxithromycin 300 mg Placebo OD / 12 weeks + Doxycycline 100 mg Placebo OD / 12 weeks

Follow-up 48 weeks (Telephone calls and visits)

4 SELECTION OF SUBJECTS

4.1 Number of subjects

As calculated in *Section 11.5 Sample size justification*, 312 subjects should be enrolled and treated with study medication at approximately 20 centres. The minimum number of subjects randomised per centre should be 6.

4.2 Inclusion criteria

Subjects meeting all of the following criteria will be considered for admission to the study:

Informed consent must be obtained for all subjects before enrolment in the study (Visit 1).

- Diagnosis of COPD (as per European Respiratory Society Consensus Statement[19]) and producing sputum most days
- Smoking history of 20 pack years or more
- ≥ 45 years of age
- Onset of COPD at ≥ 40 years of age
- FEV_1 ≤ 70% of predicted (predicted values from Ph. H. Quanier et al Eur Resp J. 1993, 6, Suppl. 16: 5-40)
- FEV_1 / FVC ratio ≤ 60%
- Reversibility of ≤15% and/or ≤ 200 mL following inhaled salbutamol (400 µg) via metered dose inhaler (MDI) with volumatic spacer, or 5 mg in 1 mL via nebuliser
- Serum IgG for *C. pneumoniae* ≥ 1:64 as measured by MIF
- At least three exacerbations of COPD in the past 2 years requiring treatment with antibiotics and/or oral corticosteroids and/or hospitalisation. These should be confirmed wherever possible by obtaining copies of medical records and/or certification by the primary care physician.
- For female subjects, the following conditions must be met:
  - Post menopausal for at least 1 year, ***or***
  - Surgically incapable of bearing children, ***or***
  - A negative pregnancy test prior to randomisation and must agree to use adequate contraceptive methods (in the investigator's opinion) for the duration of study treatment

4.3 Exclusion criteria

Subjects presenting with any of the following will not be included in the study:

- Pulmonary disease other than COPD including active pulmonary tuberculosis, bronchiectasis, interstitial lung disease, bronchial carcinoma or metastatic malignancy in the lungs
- An acute exacerbation of COPD in the 4 weeks before randomisation (such patients may be enrolled ≥ 4 weeks after resolution of an exacerbation)
- Change in stable corticosteroid therapy (inhaled or oral) during the 4 weeks before randomisation (Visit 2) (such patients may be enrolled after corticosteroid therapy has been restabilised for ≥ 4 weeks)
- Long term treatment with > 10 mg/day of prednisolone or equivalent
- Likelihood of requiring recurrent antibiotic therapy for other chronic conditions (e.g. chronic sinusitis or recurrent urinary tract infections)
- Treatment with antibiotics in the 4 weeks before randomisation (Visit 2) (such patients may be enrolled ≥ 4 weeks after ceasing antibiotics)
- Pregnancy (demonstrated by serum pregnancy test)
- Breast-feeding
- History of hypersensitivity to the study medications or rescue medications (macrolide, tetracycline, beta-lactam, sulfamethoxazole; trimethoprim) or to drugs with similar chemical structures
- Subjects who are receiving treatment with medications known to have potential drug interactions with the study medicines and/or macrolides/tetracyclines, in general, including but not limited to:

  Ergot alkaloid derivatives, terfenadine, cisapride, astemizole, pimozide, cholinesterase inhibitors (e.g. tacrine, donepizil, physostigmine), ketamine, disopyramide, quinidine, ketoconazole or itraconazole.
- Likelihood of requiring treatment during the study period with drugs not permitted by the study protocol (see *Section 6.2 Prior and concomitant treatments*)
- Treatment with any investigational drug in the 4 weeks before randomisation (Visit 2)
- Clinically relevant cardiovascular, hepatic, neurologic, endocrine, or other major systemic disease making implementation of the protocol or interpretation of the study results difficult
- Patients with a known long QT syndrome or QTc > 450 ms, sick sinus syndrome, bradycardia (<50 beats per minute) or severe hypokalaemia
- Patients diagnosed with epilepsy
- History of drug or alcohol abuse
- Impaired hepatic function at visit 1 safety blood screen, as shown by any of the following:

  - AST (SGOT) or ALT (SGPT) ≥ 2 times the upper limit of normal (ULN)

  - Alkaline phosphatase ≥ 1.25 times the upper limit of normal (ULN)

  - Bilirubin > 2 times the upper limit of normal (ULN) (Gilbert's Syndrome excepted)

  - Albumin < 30 g/L

- Impaired pancreatic function at visit 1 safety blood screen as shown by:
    - Amylase or Lipase > upper limit of normal (ULN)
- Impaired renal function as shown by creatinine clearance ≤ 20 mL/min (creatinine clearance will be estimated using the Cockcroft and Gault formula (See **Appendix A**)) at visit 1 safety blood screen
- Mental condition rendering the subject unable to understand the nature, scope, and possible consequences of the study
- Subject unlikely to comply with protocol, e.g., uncooperative attitude, inability to return for follow-up visits, and unlikelihood of completing the study

Any waiver of these inclusion and exclusion criteria must be approved by the investigator and the sponsor on a case-by-case basis prior to enrolling the subject. This must be documented by both the sponsor and the investigator.

No subject will be randomised into this study more than once.

5 STUDY TREATMENTS

5.1 Details of study treatments

The following treatments will be used in the study:

Drug:	Roxithromycin
Formulation:	Tablet containing 300 mg
Manufacturer:	Hoechst Marion Roussel
Drug:	Doxycycline
Formulation:	Tablet containing 100 mg
Manufacturer:	Douglas Manufacturing, a division of Douglas Pharmaceuticals Ltd., Lincoln, New Zealand

Identical placebo tablets will be provided.

5.2 Dosage schedule

All subjects will receive 1 tablet of roxithromycin 300 mg or matching placebo daily and 1 tablet doxycycline 100 mg or matching placebo once a day. Roxithromycin will be taken on an empty stomach (at least 15 minutes before or 3 hours after meals), and doxycycline will be taken with food or milk.

The first dose will be taken at the time of Visit 2.

5.3 Treatment assignment

The study medication will be administered only to subjects included in this study following the procedures set out in the study protocol.

A sequence of subject numbers will be assigned to each study centre. All subjects who have signed an informed consent document will receive a subject number (A number), applied in chronological ascending order at each site.

When a subject has satisfied all entry criteria and is randomised to treatment they will receive a randomisation number (B number), applied in ascending order from the drug numbers supplied to the site.

Subjects withdrawn from the study retain their subject number and their randomisation number, if already given. New subjects must always be allotted a new subject number and, if randomised, a new randomisation number.

The complete randomisation schedule will be stored with Hoechst Marion Roussel Australia.

5.4 Blinding, packaging, and labelling

The study medication will be packed by Hoechst Marion Roussel in bottles. All subjects will be instructed to take 1 tablet from Bottle A in the morning together with one tablet from Bottle B.

The bottle labels will contain the following information:

- Randomisation number
- Number of tablets
- Dose
- Special instructions
- Batch number
- Storage conditions
- The words << For clinical trial use only >>
- Expiration date
- Address of the sponsor

Bottle 1 (***Bottle A***) of roxithromycin or matching placebo will contain 19 (2 weeks +5 days) tablets.
Bottle 2 *(Bottle A)* of roxithromycin or matching placebo will contain 33 (4 weeks + 5 days) tablets.
Bottle 3 *(Bottle A*) of roxithromycin or matching placebo will contain 42 (exactly 6 weeks) tablets.

Bottle 1 (***Bottle B***) of doxycycline or matching placebo will contain 19 (2 weeks +5 days) tablets.
Bottle 2 *(Bottle B)* of doxycycline or matching placebo will contain 33 (4 weeks + 5 days) tablets.
Bottle 3 *(Bottle B*) of doxycycline or matching placebo will contain 42 (exactly 6 weeks) tablets.

Bottle 1 (A+B) – Dispensed at Visit 2 (randomisation), returned at Visit 3 (2 weeks post randomisation)

Bottle 2 (A+B) – Dispensed at Visit 3 (2 weeks post randomisation), returned at Visit 4 (6 weeks post randomisation)

Bottle 3 (A+B) – Dispensed at Visit 4 (6 weeks post randomisation), returned at Visit 5 (12 weeks post randomisation)

In addition to the study medication, the investigator will receive a set of sealed envelopes, one envelope for each randomisation number. An identical set of sealed envelopes will be held by the sponsor. These envelopes contain information on the subject's study medication and are to be opened only under circumstances in which it is medically imperative to know what the subject is receiving. The randomisation envelopes are not to be opened by the investigator at the end of the study. All envelopes will be collected by the sponsor at the end of the study.

Details of emergency unblinding procedures are given in Section 10.2 Emergency identification of study medication.

5.5 Supplies and accountability

The investigator or pharmacist will inventory and acknowledge receipt of all shipments of study medication. All study medication must be kept in a locked area with access restricted to designated study personnel. The study medication must be stored in accordance with the manufacturer's instructions. The investigator or pharmacist will also keep accurate records of the quantities of study medication dispensed, used, and returned by each subject. The site monitor will periodically check the supplies of study medication held by the investigator or pharmacist to ensure accountability of all study medication used. At the conclusion of the study, all unused study medication and all medication containers will be returned to the sponsor unless other arrangements have been approved by the sponsor. The sponsor will assure that a final report of drug accountability to the unit dose level is prepared and maintained by the investigator.

5.6 Compliance

Subjects will be instructed to bring their study medication bottles to visits 3, 4 and 5. Compliance will be assessed by tablet counts.

6 PRIOR AND CONCOMITANT ILLNESSES AND TREATMENTS

6.1 Prior and concomitant illnesses

Prior, significant illnesses will be documented in the case report form.

Additional illnesses present at the time informed consent is given are regarded as concomitant illnesses and will be documented on the appropriate pages of the case report form.

Illnesses first occurring or detected during the study, and worsening of a concomitant illness during the study, are to be regarded as adverse events and must be documented as such in the case report form.

6.2 Prior and concomitant treatments

Concomitant therapy for all patients randomised into the study (Visit 2), will be documented in the case report form, including all medications from Visit 1 onwards.

All additional treatments being taken by the subjects on entry to the study or at any time during the study are regarded as concomitant treatments and must be documented on the appropriate pages of the case report form.

The following concomitant treatments *are permitted* during this study:

- Inhaled and/or oral beta-adrenergic agonists e.g. salbutamol, terbutaline, eformoterol, salmeterol.
- Inhaled anticholinergic agents e.g. ipratropium bromide.
- Inhaled corticosteroids e.g. beclomethasone dipropionate, fluticasone propionate, budesonide.
- Oral corticosteroids, if the dose has not changed (≤ 10 mg/day equivalent prednisolone), in the 4 weeks prior to randomisation (Visit 2).
- Beta-lactam antibiotics (e.g. amoxycillin/potassium clavulanate, cefaclor, cefuroxime, cephalexin, penicillin, amoxycillin), or sulfamethoxazole; trimethoprim for exacerbations of COPD or for pneumonia for periods of up to 10 days at a time.
- Antacids are permitted but must not be taken within 2 hours (before or after) of taking doxycycline (Bottle B)

The following medications *are permitted*, but must be monitored:

Interactions between macrolide and/or tetracycline antibiotics and some other drugs have been reported. Additional monitoring as described below, is necessary at pre-therapy (Visit 1), on-therapy (Visit 3 and 4) and end of therapy (Visit 5) visits for the following drugs:

- Warfarin - PT and/or INR
- Digoxin - serum levels
- Theophylline - serum levels

The following concomitant treatments *are not permitted* during this study:

- The following antibiotics should not be used during the study unless this is absolutely necessary for the subject's welfare: macrolides, quinolones, tetracyclines, chloramphenicol or any antibiotics active against *C. pneumoniae.*
- Ergot alkaloid derivatives, terfenadine, cisapride, astemizole, pimozide, cholinesterase inhibitors (e.g. tacrine, donepizil, physostigmine), ketamine, disopyramide, quinidine, ketoconazole or itraconazole.

7 STUDY PROCEDURES AND SCHEDULE

7.1 Overview of data collection

Efficacy Data

The primary outcomes for efficacy are the frequency of moderate and severe acute exacerbations, and the severity of all exacerbations, of COPD.

An ***acute exacerbation*** is defined as at least 2 out of 3 of the following, on 3 consecutive days or more:

(see **Appendix B** *for exacerbation scale)*

- Change in sputum production of 1 point or more
- Change in colour (purulence) of sputum of 1 point or more

(e.g. white to light yellow, light yellow to dark yellow/green, but not from clear to white)

- Change in breathlessness of 2 points or more

Exacerbation severity is operationally defined as:

Mild – self-managed by the patient at home (e.g. increase in bronchodilator and/or non prescription medication (OTC) use)

Moderate – requiring treatment with antibiotic and/or an increase in dose of, or initiation of corticosteroids by a medical practitioner (e.g. family physician, specialist, hospital outpatients or Accident and Emergency Unit

Severe – resulting in admission to hospital or death due to an exacerbation of COPD

The secondary outcomes for efficacy are:

- Frequency of all exacerbations (any severity)
- FEV_1 and FVC.
- Scores on the Chronic Respiratory Disease Questionnaire (CRQ).
- Titres of IgG and IgA antibodies for *C. pneumoniae.*
- Eradication of *C. pneumoniae* in the sputum as determined by sputum PCR
- Reduction in secretory IgA to *C. pneumoniae* in sputum
- Eradication of *C. pneumoniae* in monocytes
- Cost effectiveness of treatment

Samples of serum and sputum will be retained and may be retested when new tests relevant to the trial's objectives become available. These may be for such things as improved markers for chronic infection with *C. pneumoniae.*

Other outcomes

- Microbiological examination of sputum will also be undertaken at selected centres to determine the effect of long term broad spectrum antibiotic therapy on the resident flora of sputum (Appendix J).

Safety Data

Safety will be assessed on the basis of:

- Physical examination
- Adverse events
- Laboratory parameters
- Electrocardiogram (ECG)

Compliance

Compliance will be assessed by counting returned study medication at Visit 3 (2 weeks), Visit 4 (6 weeks) and Visit 5 (12 weeks).

Subjects will be considered non-compliant from the perspective of the per-protocol analysis if they miss > 20% of all medication.

Economic Outcomes

Health resource usage effects of treatment will be evaluated by comparing all treatment groups in terms of:

- Number of hospitalisations
- Number of visits to medical practitioners, A&E or other health care professionals
- Alterations to drug usage
- Details of laboratory tests or other procedures

where these are related to COPD.

This information will be collected using the subject Daily Diary Card.

7.2 Description of study days

7.2.1 Prestudy screening

Visit 1 (Week –2-4): Screening Visit.

Subjects will be asked to withhold inhaled beta-agonists for relevant periods (4 hours, long acting for 12 hours and salmeterol for 24 hours) and inhaled ipratropium bromide for 6 hours prior to the study visit (and on subsequent study visits when spirometry is to be conducted).

The following assessments will be made:

- Informed Consent will be signed
- Eligibility criteria reviewed (inclusion / exclusion)
- Demographics (including height and weight)
- Medical history, including number of exacerbations in previous 2 years and details of risk factors (prior hospitalisations, pack years of smoking, inhaled steroid use)
- Spirometry (FEV_1 and FVC and FEV_1 reversibility) if bronchodilators were not withheld, reconfirm at Visit 2 (prior to randomisation) if all other criteria are met.

  (As per European Respiratory Society Guidelines ERS Working Party. Standardised Lung Function Testing – Official Statement of ERS - Eur. Respir. J. 1993; 6 (Suppl. 16: 1-100))
- Pregnancy test (if applicable)
- Blood will be collected for safety screen (haematology, biochemistry), monocyte PCR and serology (if IgG positive, the patient should continue to visit 2)
- Collection of sputum (spontaneous or induced) for sIgA antibodies and PCR for *C. pneumoniae* and for microbiological evaluation (at selected centres)
- Provide Daily Diary Card if patient eligible as per known above criteria and the use explained (for training purposes only – the data from this pre-randomisation Diary Card will not be used)

7.2.2 Study days

Visit 2 (Week 0): Randomisation

Visit 2 must occur within 2-4 weeks of Visit 1 if IgG serology for *C. pneumoniae* is positive and the subject satisfies the rest of the eligibility criteria for the study. If the subject has an exacerbation after Visit 1 and before randomisation, Visit 2 may be delayed until 12 weeks after Visit 1. If the delay is any longer, Visit 1, especially the baseline serology (IgG), must be repeated.

The following assessments will be performed:

- Recheck inclusion/exclusion criteria and ability to complete Daily Diary Card
- Validate at least three exacerbations of COPD in the past 2 years requiring treatment with antibiotics and/or oral corticosteroids and/or hospitalisation. These must be confirmed where possible by reviewing medical records and/or certification by the primary care physician.
- Quality of Life Questionnaire (CRQ)
- Physical examination
- 12 Lead Electrocardiogram (ECG)
- Chest X-ray

- Spirometry (FEV_1 and FVC and FEV_1 reversibility) if not conducted at Visit 1
- Adverse events and concomitant medication will be recorded
- Another Diary Card will be issued after the previous Diary Card is checked
- If an exacerbation of COPD has occurred, the patient cannot be randomised until at least four weeks after it has resolved.
- A Patient ID Card will be issued to be shown to their doctor / hospital whenever they seek attention for acute exacerbations of COPD. The card will indicate that if they require treatment with antibiotics they should be treated with beta-lactam antibiotics or sulfamethoxazole/trimethoprim. The antibiotics which should ***not be used*** (unless absolutely necessary) will also be listed on the card. These antibiotics are macrolides, fluoroquinolones, tetracyclines and chloramphenicol.
- The subject will be assigned a randomisation number. The number will be allocated in sequence and the subject will receive the next available number and the corresponding medication labelled box (Bottle 1, A+B). The subject will be issued with 2 weeks supply of the study medicine. The subject will be instructed to take one tablet of roxithromycin or matching placebo daily at least 15 minutes before food or three hours after food, and one tablet of doxycycline or matching placebo daily with food or milk. The patient should take the first tablets during the evening of the day of consultation (i.e.1 tablet roxithromycin (or placebo) plus 1 tablet doxycycline (or placebo)).
- Visit 3 should be scheduled in 2 weeks (± 3 days)

All subsequent visits as far as possible should be conducted at the same time of day as the run-in visit.

Visit 3 (Week 2 ± 3 days): On Treatment

The diary card will be collected along with any unused medication.

- Blood will be collected for safety screen (haematology, biochemistry)
- 12 Lead Electrocardiogram (ECG)
- Adverse events and concomitant medication will be recorded
- COPD exacerbations will be recorded
- Another diary card will be issued after the previous Diary Card is checked
- A further 4 week supply of study medication will be dispensed (Bottle 2, A+B) following a compliance check of previous medication.
- Schedule visit 4, Telephone 1 and Week 4 safety blood test

Telephone contact 1 (Week 4 ± 3 days): On Treatment

Safety Monitoring (Week 4 ± 3 days, Week 9 ± 3 days (On Treatment), and Week 16 (follow up)

Blood will be collected for safety testing (haematology, biochemistry)

Visit 4 (Week 6 ± 3 days): On Treatment.

The diary card will be collected along with any unused medication.

The following assessments will be performed:

- 12 Lead Electrocardiogram (ECG) (if abnormal at visit 3)
- Adverse events and concomitant medication will be recorded
- COPD exacerbations will be recorded
- Another diary card will be issued after the previous Diary Card is checked
- A further 6 week supply of study medication will be dispensed (Bottle 3, A+B) following a compliance check of previous medication
- Schedule visit 5

Telephone Contact 2 (Week 9 ± 3 days): On Treatment

Visit 5 (Week 12 ± 3 days): End of Treatment

This visit occurs at the end of the treatment period. The diary card will be collected along with any unused medication and empty containers.

The following assessments will be performed:

- Physical examination
- Quality of Life Questionnaire (CRQ)
- Spirometry (FEV_1 and FVC)
- 12 Lead Electrocardiogram (ECG)
- Blood will be collected for safety screen (haematology, biochemistry), monocyte PCR and serology
- Adverse events and concomitant medication will be recorded
- COPD exacerbations will be recorded
- Collection of sputum (spontaneous or induced) for sIgA antibodies/PCR for *C. pneumoniae* and for microbiological evaluation
- Another diary card will be issued after the previous Diary Card is checked
- Schedule visit 6 and Telephone 3 and 4

Visit 6 (24 weeks ± 5 days) and visit 8 (48 weeks ± 5 days) Follow-up visits.

Following the end of treatment there will be follow-up visits every 12 weeks. The first follow-up visit (Visit 6) will be 12 weeks **after the end of treatment** .

The following assessments will be performed at each of these visits:

- Adverse events and concomitant medication will be recorded
- COPD exacerbations will be recorded
- Another diary card will be issued after the previous Diary Card is checked
- 12 Lead Electrocardiogram (ECG) will be performed at visit 6 if abnormal at previous visit (ECG is not required at visit 8)
- Schedule next visit and telephone calls

Visit 7 (Week 36 ± 5 days): Follow-up & Visit 9 (Week 60): (48 weeks after completion of study medication)

The following assessments will be performed:

- Quality of Life Questionnaire (CRQ)
- Spirometry (FEV_1 and FVC)
- Serum for *C. pneumoniae* serology
- Blood for monocyte PCR
- Provision of sputum (spontaneous or induced) for sIgA antibodies for *C. pneumoniae* and for microbiological evaluation (at selected centres)
- Adverse events and concomitant medication will be recorded
- COPD exacerbations will be recorded
- Diary Card will be checked
- Schedule visit 8 and telephone calls (visit 7 only)

Telephone Assessments.

The study site will telephone the subject at 4, 9, 16, 20, 28, 32, 40, 44, 52 and 56 weeks from **start of treatment** (± 5 days). If necessary, more frequent telephone calls can be made. The study site will determine if the subject has had any exacerbations, any changes in concomitant medication or any problems since the last visit.

Exacerbations/Unscheduled contacts

If a subject believes that they are experiencing an exacerbation at any time during the study they must contact the investigator and advise the details of their symptoms. If the exacerbation is judged to be of greater than mild severity this will require a visit to the subject's regular doctor or admission for evaluation and probably prescription of appropriate medications. Regimens for administration of medications prescribed during an exacerbation are defined in **Appendix C**.

7.2.3 Liaison throughout Study Period

Contact will be maintained between the study nurses / investigators, general medical practitioners and subjects throughout the study period.

- Site staff should contact the subject's General Practitioner in first instance (at Visit 2) to detail study importance regarding follow-up, exacerbations, treatment etc. It may be necessary to contact the GP to confirm exacerbations in the previous two years to confirm inclusion criteria.
- Subjects should be instructed to telephone the study nurse or investigator if they suspect they are suffering an exacerbation
- The study nurse or investigator should telephone the General Practitioner regularly e.g. 4 weekly to assist with documenting exacerbations and treatment
- The General Practitioner or practice nurse should be asked to telephone the study nurse or investigator if an exacerbation occurs

7.3 Methods of data collection

7.3.1 Efficacy data

Exacerbations

The number of exacerbations will be determined from the Daily Diary Card and investigator assessment.

An acute exacerbation is defined as at least 2 out of 3 of the following, on 3 consecutive days or more:

(see **Appendix B** *for exacerbation scale)*

- Change in sputum production of 1 point or more
- Change in colour (purulence) of sputum of 1 point or more
  (e.g. white to light yellow, light yellow to dark yellow/green, but not from clear to white)
- Change in breathlessness of 2 points or more

Exacerbation severity is operationally defined as:

Mild	– self-managed by the patient at home (increase in bronchodilator and non prescription medication (OTC) use)
Moderate	– requiring treatment with antibiotic and/or an increase in dose of, or initiation of corticosteroids by a family physician or as a hospital outpatient
Severe	– resulting in admission to hospital or death due to COPD

Quality of Life Questionnaire

The Chronic Respiratory Disease Questionnaire will be administered **(See Appendix D).** This will always be administered before any other assessments or investigations at the visit.

Forced Expiratory Volume in One Second (FEV_1) and Forced Vital Capacity (FVC)

Spirometry will be performed according to the guidelines of the European Respiratory Society **(See Appendix E).**

Symptom Scores

The symptoms of sputum quantity and purulence and breathlessness will be scored on a Daily Diary Card using the symptom scales in **Appendix B.**

Serology

IgG and IgA antibodies for *C. pneumoniae* will be measured by microimmunofluorescence **(see Appendix F).**

Sputum

C. pneumoniae will be evaluated in sputum using PCR and secretory IgA from all patients at all centres **(See Appendix H).**

Monocytes

C. pneumoniae will be evaluated in peripheral blood mononuclear cells using PCR from all patients at all centres.

7.3.2 Safety data

Medical History

A full history will be obtained at the screening visit.

Physical examination

A full physical examination will be performed at randomisation (Visit 2) and at the end of treatment (Visit 5).

Adverse Events

Adverse events observed by the investigator or reported by the subject will be documented as described in *Section 8 Adverse events*.

12 Lead Electrocardiogram (ECG)

At randomisation (Visit 2), on-therapy (Visit 3) and end of therapy (Visit 5) a 12 Lead ECG will be performed for all patients. If the ECG at Visit 3 (on-therapy) is abnormal, this should be repeated at Visit

4 (on-therapy), otherwise, the next ECG will be done at Visit 5 (end of therapy). If the results of the ECG at Visit 5 are abnormal, an additional ECG should be performed at Visit 6.

Whenever possible, 12 Lead ECGs will be performed at 1 to 3 hours after drug intake and be calibrated to 2 cm/mV.

ECG recordings will be printed in duplicate and include:

1) Protocol number 2) Subject and (if applicable) Randomisation number 3) Subject initials

4) Date and time of ECG

One copy will be kept at the study site. The copy that is made available to the sponsor will be the one that is read at the study site.

The corrected QT (QTc) interval will be calculated using Bazett's formula (Bazett HC. An analysis of the times relations of electrocardiograms. Heart 1920;7:353-70)

The timing of the ECGs and study medication intake will be recorded on the case report form.

Laboratory Data

The laboratory parameters listed in **Appendix H** will be measured. If abnormalities are detected which are considered to be attributable to the study medication they must be followed up until the values return to normal.

Laboratory abnormalities considered to be clinically relevant will be reported as adverse events and will be monitored accordingly (See *Section 8 Adverse events*).

7.3.3 Pharmacokinetic/Pharmacodynamic data

Not applicable

7.3.4 Quality-of-life

See Quality of Life Questionnaire (**Appendix D**).

7.3.5 Pharmacoeconomic data

Pharmacoeconomic data will be collected, with the help of patient Daily Diary Cards, as follows:

- Time spent in hospital
- Out-patient visits
- Visits to Accident & Emergency
- Home visits from health care professionals
- Visits to General Practitioners
- Details of laboratory tests or other procedures

- Changes in medication usage including OTC drugs
- Changes in other COPD therapies and equipment e.g. oxygen, nebulisers.

To determine indirect costs related to COPD, subjects will record on their Daily Diary Card

- Loss of time from work (days)

The employment status of each subject will be determined prior to randomisation.

If patients are withdrawn (*Section 9.1 Withdrawal of subjects)* every effort should be made to continue to collect resource use data for the full 48 week follow up period.

8 ADVERSE EVENTS

8.1 Definitions

8.1.1 Adverse event

The term **adverse event** covers any sign, symptom, syndrome, or illness that appears or worsens in a subject during the period of observation in the clinical study and that may impair the well being of the subject. The term also covers laboratory findings or results of other diagnostic procedures that are considered to be clinically relevant (e.g., that require unscheduled diagnostic procedures or treatment measures, or result in withdrawal from the study).

The adverse event may be:

- A new illness
- Worsening of a sign or symptom of the condition under treatment or of a concomitant illness
- An effect of the study medication
- An effect of a comparator drug
- Unrelated to participation in the clinical study
- A combination of one or more of these factors

Thus, no causal relationship with the study medication is implied by the use of the term "adverse event".

Surgical procedures themselves are not adverse events; they are therapeutic measures for conditions that require surgery. The condition for which the surgery is required may be an adverse event. Planned surgical measures permitted by the study protocol and the condition(s) leading to these measures are not adverse events.

Adverse events fall into the categories non-serious and serious (see *Section 8.1.2 Serious adverse events)*

8.1.2 Serious adverse events

A serious adverse event (AE) is any adverse event that at any dose of the study medication or at any time during the period of observation:

- Results in death
- Is life-threatening[1]
- Requires inpatient hospitalisation or prolongation of existing hospitalisation
- Results in persistent or significant disability/incapacity[2]
- Is a congenital anomaly/birth defect
- Is medically important[3]

[1]This term means that the patient was at immediate risk of death at the time of the AE; it does not refer to an AE which hypothetically might have caused death if it were more severe.

[2]This term means that there is a substantial disruption of a person's ability to carry out normal life functions.

[3]Medical and scientific judgement should be exercised in deciding whether other AEs may be considered serious because they jeopardise the patient or may require intervention to prevent one of the other outcomes listed in the definition above. Examples of such events are intensive treatment in an emergency room or at home for allergic bronchospasm; blood dyscrasias or convulsions that do not result in hospitalisation; or development of drug dependency or drug abuse. The List of Critical Terms (1998 adaptation of WHO Adverse Reaction Terminology Critical Terms List) should be used as guidance for AEs which may be considered serious because they are medically important.

Cases involving cancer as an AE/ADR should be reported as serious using the criterion medically important.

Cases of overdose with an AE that meets one of the criteria given above should of course be reported as serious. In addition, it is HMR policy that cases from clinical trials and post-marketing surveillance studies in which a "significant overdose" was taken by a patient and a non-serious AE occurred or *no* AE occurred, are to be reported to the company in an expedited way on a serious adverse event (SAE) form. This is so that HMR is notified of this important information on the consequences of overdose without delay. This information may be used to update the overdose section of the product information and to provide advice in the future on the management of patients who have taken an overdose.

Clarification on the difference in meaning between "severe" and "serious"

The term "severe" is often used to describe the intensity (severity) of a specific event (as in mild, moderate, or severe myocardial infarction); the event itself, however, may be of relatively minor medical significance (such as severe headache). This is not the same as "serious," which is based on patient/event outcome or action criteria usually associated with events that pose a threat to a patient's life or functioning. Seriousness (not severity) serves as a guide for defining regulatory reporting obligations.

8.1.3 Alert terms

No special events are subject to reporting as alert terms in this study.

8.2 Period of observation

For the purposes of this study, the period of observation for adverse events extends from the time the subject gives informed consent until 2 weeks after treatment is completed, or 12 months after treatment is completed if the investigator considers the event is related to the study medication.

If the investigator considers it necessary to report an adverse event in a study subject after the end of the period of observation, he or she should contact the sponsor to determine how the adverse event should be documented and reported.

8.3 Documentation and reporting of adverse events by investigator

All adverse events that occur after the subject has signed the informed consent document must be documented on the pages provided in the case report form in accordance with the "Instructions for the completion of adverse event reports in clinical studies" in the investigator site file. The adverse events must also be recorded in the subject's medical records.

- All serious adverse events must also be documented on "Serious adverse event" forms.

Every attempt should be made to describe the adverse event in terms of a diagnosis. If appropriate, component symptoms should also be listed below the diagnosis. If only nonspecific signs or symptoms are present, these should each be recorded as a diagnosis.

For each event occurring after ***the first dose of study medication/randomisation***, the investigator will classify whether the event is to be considered treatment emergent and whether there is a reasonable possibility that the event was associated with the use of the study medication.

An adverse event that occurs during the study after ***the first dose of study medication/randomisation*** will be considered as treatment-emergent if, (1) it was not present at ***the time of the first dose of study medication/randomisation*** and is not a chronic condition that is part of the patient's medical history, or (2) it was present at ***the time of the first dose of study medication/randomisation*** or as part of the patient's medical history, but its intensity (severity or frequency) has worsened after ***the first dose of study medication/randomisation***.

Adverse events that fulfil the criteria for seriousness (*Section 8.1.2 Serious adverse events)*

8.4 Immediate reporting by investigator to sponsor

Serious adverse events and adverse events that comply with any alert terms must be documented on "Serious adverse event" forms and supplied to the sponsor within 24 hours or at the latest on the following working day. The investigator must also inform the site monitor in all cases. The sponsor will ensure that all legal reporting requirements are met.

The initial report must be as complete as possible, including details of the current illness and (serious) adverse event and an assessment of the causal relationship between the event and the study medication.

Copies of the case report form pages containing the following information (if not already supplied to the sponsor) must be sent with the "Serious adverse event " form:

- Demography
- Medical and surgical history
- Previous and concomitant medication
- Study medication administration record

The forms documenting all non-serious adverse events that have occurred up to the time of occurrence of a serious adverse event, even if still incomplete, must also be sent with the "Serious adverse event" form.

Information not available at the time of the initial report (e.g. an end date for the adverse event or laboratory values received after the report) must be documented on a follow-up "Serious adverse event" form that carries the number(s) of the initial report(s).

The "Instructions for the completion of adverse event reports in clinical studies" give more detailed guidance on the reporting of serious adverse events, adverse events that comply with alert terms, and adverse events initially reported as non-serious that become serious.

The adverse events leading to death must be reported in accordance with the above, and the "In case of death" page in the case report form must be supplied to the sponsor with the "Serious adverse event" form.

9 WITHDRAWALS

9.1 Withdrawal of subjects

Subjects may be withdrawn from study medication for the following reasons:

- At their own request or at the request of their legally authorised representative
- If, in the investigator's opinion, continuation in the study would be detrimental to the subject's well-being
- At the specific request of the sponsor

Subjects must be withdrawn from study medication under the following circumstances:

- Impaired hepatic function during treatment as shown by an increase to ≥ 3 times the upper limit of normal of one or more of AST (SGOT), ALT (SGPT) and total bilirubin or, by an increase to ≥ 1.25 times the upper limit of normal of alkaline phosphatase.
- The occurrence of alarming adverse events that may be related to study medication
- Pregnancy (every attempt must be made to follow up subjects who become pregnant to determine the outcome of the pregnancy)
- QTc interval ≥500 ms
- Impaired renal function creatinine clearance ≤ 20 mL/min
- Impaired pancreatic function, amylase or lipase > 2 times the upper limit of normal (ULN)

In all cases, the reason for withdrawal must be recorded in the case report form and in the subject's medical record. The subject must be followed up to establish whether the reason was an adverse event, and, if so, this must be reported in accordance with the procedures in *Section 8 Adverse events)*

Subjects withdrawn from study medication should remain in the study for the full assessment period wherever possible.

As far as possible, all examinations scheduled for the final study day must be performed on all subjects who receive study medication but do not complete the study according to protocol (see *Section 7.2 Description of study days).*

As far as possible, health resource use data should be collected for the full 48 week follow up period for withdrawn patients (*Section 7.3.5 Pharmacoeconomic data)*.

The investigator must make every effort to contact subjects lost to follow-up.

9.2 Replacement of subjects

Subjects will not be replaced.

10 EMERGENCY PROCEDURES

10.1 Emergency sponsor contact

In emergency situations, the investigator should contact the sponsor by telephone at the number listed on the title page of the protocol.

10.2 Emergency identification of study medication

If it is medically imperative to know what study medication the subject is receiving, the investigator or authorised person should open the randomisation envelope. The investigator or the person who breaks the blind must record the date and the reasons for doing so in the case report form, in the subject's medical record, and on the randomisation envelope. In such cases, treatment with the study medication must be stopped and the sponsor contacted immediately to determine whether the subject should be withdrawn from the study. Whenever possible, the sponsor should be contacted before the blind is broken.

10.3 Emergency treatment

During and following a subject's participation in the trial, the investigator/institution should ensure that adequate medical care is provided to a subject for any adverse events, including clinically significant laboratory values, related to the trial. The investigator/institution should inform a subject when medical care is needed for intercurrent illness(es) of which the investigator becomes aware.

11 STATISTICAL PROCEDURES

11.1 Analysis variables

11.1.1 Primary outcome variables

The primary outcome variables for the study will be the frequency of moderate and severe exacerbations, and the severity of all exacerbations, of COPD reported by each patient over a 48 week period and confirmed by investigator assessment.

An ***acute exacerbation*** is defined as 2 out of 3 of the following, on 3 consecutive days or more:

(see **Appendix B** *for exacerbation scale)*

- Change in sputum production of 1 point or more
- Change in colour (purulence) of sputum of 1 point or more
  (e.g. white to light yellow, light yellow to dark yellow/green, but not from clear to white)
- Change in breathlessness of 2 points or more

Exacerbation severity is defined as:

Mild	– self-managed by the patient at home (increase in bronchodilator and non-prescription medication (OTC) use)
Moderate	– requiring treatment with antibiotic and/or an increase in the dose of or initiation of corticosteroids by a medical practitioner (investigator, family physician or at hospital outpatients)
Severe	– resulting in admission to hospital or death, due to COPD

11.1.2 Secondary outcome variables

The secondary efficacy outcomes will be:

The number of exacerbations of COPD reported by each patient over the 24 week period after treatment

FEV_1 and FVC measured over a 60 week period

Scores on the Chronic Respiratory Disease Questionnaire (CRQ) measured over a 60 week period

Titres of IgG and IgA antibodies for *C. pneumoniae*

Eradication of *C. pneumoniae* in the sputum as determined by sputum PCR

Reduction in secretory IgA to C. *pneumoniae* in sputum

Eradication of *C. pneumoniae* from monocytes

Pharmacoeconomics

11.2 Study populations

Patients who are not exposed to study medication will not be considered evaluable for the assessment of efficacy and safety. The remaining randomised patients will be identified as the intention to treat population. These patients will be classified into one of two categories:

Protocol correct (per protocol)

Patients with no major protocol violations that would significantly affect the interpretation of efficacy.

Not protocol correct

Patients with major protocol violations that would significantly affect the interpretation of efficacy. These are defined as non-compliant, did not complete 60 weeks of trial (i.e. lost to follow up), major change in maintenance medications, patients prescribed macrolides, quinolones or tetracyclines during study.

11.3 Statistical methods

A description of the demographic and baseline characteristics will be given for each treatment group and the groups checked for comparability. All relevant individual subject demographic and baseline data will be presented for all randomised subjects (sorted by treatment group and centre).

Statistical analysis for all of the primary and secondary efficacy parameters will be performed at the end of the 60 week study period in comparison with baseline. Analysis of variance models will be pursued to test for differences in numbers of exacerbations between 3 treatment groups. Covariates in the models will include centre, age and sex. Treatment by centre and treatment by covariate interactions will also be inspected. Point estimates and associated confidence intervals for differences between treatments will be presented.

For daily diary card symptom score data, repeated measures models will be used to estimate the overall impact of treatment during the entire study period.

11.4 Interim analysis

No interim analysis is planned for this study.

11.5 Sample size justification

The mean number of moderate and severe exacerbations per year is estimated to be 2.5 with a standard deviation of 1.0 in each treatment group. An analysis of variance based F-test would require 83 subjects in each treatment group to have 90% power to detect a difference in the exacerbation rate of 0.5 per year, (i.e. under the alternate hypothesis of 2.5 exacerbations per year in the control group and 2 exacerbations per year in each of the active treatment groups) at the 5% significance level. Thus a total of 249 subjects would be required. If the drop out rate was approximately 20% one would need to recruit 312 subjects to ensure sufficient numbers of completers.

The required number of patients will be reviewed when half the planned number of patients have been randomised (156 patients). The power of the study to detect a difference in exacerbation rate of 0.5 per year depends on the baseline exacerbation rate. If this is substantially different from 2.5 per year, the total number of patients required will be adjusted in order to provide an adequate chance of detecting a significant difference if one exists.

12 ETHICAL AND LEGAL ASPECTS

12.1 Good clinical practice

The procedures set out in this study protocol, pertaining to the conduct, evaluation, and documentation of this study, are designed to ensure that the sponsor and investigator abide by the principles of the good clinical practice (GCP) guidelines of ICH Harmonised Tripartite Guideline, "Good Clinical Practice: Consolidated Guideline", participating countries own GCP Guidelines (i.e. Guidelines for Good Clinical Research Practice (GCRP) in Australia), and the ethical principles laid down in the current revision of the Declaration of Helsinki. The study will also be carried out in keeping with local legal and regulatory requirements.

12.2 Delegation of investigator responsibilities

The investigator should ensure that all persons assisting with the trial are adequately informed about the protocol, any amendments to the protocol, the study treatments, and their trial-related duties and functions.

The investigator should maintain a list of sub-investigators and other appropriately qualified persons to whom he or she has delegated significant trial-related duties.

12.3 Subject information and informed consent

Before being admitted to the clinical study, the subject must consent to participate after the nature, scope, and possible consequences of the clinical study have been explained in a form understandable to him or her.

An informed consent document that includes both information about the study and the consent form will be prepared and given to the subject. This document will contain all locally required elements and Hoechst Marion Roussel requirements. The document must be in a language understandable to the subject and must specify who informed the subject. Where required by local law, the person who informs the subject must be a physician.

After reading the informed consent document, the subject must give consent in writing. The subject's consent must be confirmed at the time of consent by the personally dated signature of the and by the personally dated signature of the person conducting the informed consent discussions.

Consent must be confirmed at the time of consent orally and by the personally dated signature of the subject. The witness and the person conducting the informed consent discussions must also sign and personally date the consent document.

A copy of the signed consent document must be given to the subject. The original signed consent document will be retained by the investigator.

The investigator will not undertake any measures specifically required for the clinical study until valid consent has been obtained.

The investigator should inform the subject's primary physician about the subject's participation in the trial if the subject has a primary physician and if the subject agrees to the primary physician being informed.

12.4 Confidentiality

Subject names will not be supplied to the sponsor. Only the subject number and subject initials will be recorded in the case report form, and if the subject name appears on any other document (e.g., pathologist report), it must be obliterated before a copy of the document is supplied to the sponsor. Study findings stored on a computer will be stored in accordance with local data protection laws. The subjects will be told that representatives of the sponsor, IEC/IRB, or regulatory authorities may inspect their medical records to verify the information collected, and that all personal information made available for inspection will be handled in strictest confidence and in accordance with local data protection laws.

The investigator will maintain a personal subject identification list (subject numbers with the corresponding subject names) to enable records to be identified.

12.5 Protocol amendments

Neither the investigator nor the sponsor will alter this study protocol without obtaining the written agreement of the other. Once the study has started, amendments should be made only in exceptional cases. The changes then become part of the study protocol.

12.6 Approval of the study protocol and amendments

Before the start of the study, the study protocol, informed consent document, and any other appropriate documents will be submitted to the independent ethics committee (IEC)/institutional review board (IRB) with a cover letter or a form listing the documents submitted, their dates of issue, and the site (or region or area of jurisdiction, as applicable) for which approval is sought. If applicable, the documents will also be submitted to the authorities, in accordance with local legal requirements.

Study medication can only be supplied to the investigator after documentation on <u>all</u> ethical and legal requirements for starting the study has been received by the sponsor. This documentation must also include a list of the members of the IEC/IRB and their occupation and qualifications. If the IEC/IRB will not disclose the names of the committee members, it should be asked to issue a statement confirming that the composition of the committee is in accordance with GCP. Formal approval by the IEC/IRB should preferably mention the study title, study code, study site (or region or area of jurisdiction, as applicable), and any other documents reviewed. It must mention the date on which the decision was made and must be officially signed by a committee member.

Before the first subject is enrolled in the study, all ethical and legal requirements must be met.

The IEC/IRB and, if applicable, the authorities must be informed of all subsequent protocol amendments, in accordance with local legal requirements. Amendments must be evaluated to determine whether formal approval must be sought and whether the informed consent document should also be revised.

The investigator must keep a record of all communication with the IEC/IRB and, if applicable, between a coordinating investigator and the IEC/IRB. This also applies to any communication between the investigator (or coordinating investigator, if applicable) and the authorities.

12.7 Ongoing information for independent ethics committee/ institutional review board

If required by legislation or the IEC/IRB, the investigator must submit to the IEC/IRB:

- Information on serious or unexpected adverse events as soon as possible

- Periodic reports on the progress of the study

12.8 Premature closure of the study

The sponsor or the investigator has the right to close this study at any time. As far as possible, this should occur after mutual consultation. The IEC/IRB must be informed, if required by legislation.

Should the study be closed prematurely, all study materials (completed, partially completed, and blank case report forms, study medication, etc.) must be returned to the sponsor, as if the study had been completed.

12.9 Record retention

The following records must be retained by the investigator for a *minimum* of 15 years (or participating countries' guidelines) after the completion or termination of the study:

- Signed informed consent documents for all subjects
- Subject identification code list, screening log (if applicable), and enrolment log
- Record of all communications between the investigator and the IEC/IRB
- Composition of the IEC/IRB (or other applicable statement as described in *Section 12.6 Approval of the study protocol and amendments*)
- Record of all communications between the investigator and sponsor (or CRO)
- List of sub-investigators and other appropriately qualified persons to whom the investigator has delegated significant trial-related duties, together with their roles in the study and their signatures
- Copies of case report forms and of documentation of corrections for all subjects
- Drug accountability records
- Record of any body fluids or tissue samples retained
- All other source documents (patient records, hospital records, laboratory records, etc.)
- All other documents as listed in Section 8 of the ICH consolidated guideline on GCP (Essential Documents for the Conduct of a Clinical Trial)

However, because of international regulatory requirements, the sponsor may request retention for a longer period of time. The investigator must therefore obtain approval in writing from the sponsor prior to destruction of any records.

Normally, these records will be held in the investigator's archives. If the investigator is unable to meet this obligation, he or she must ask the sponsor for permission to make alternative arrangements. Details of these arrangements should be documented.

12.10 Liability and insurance

The sponsor has subscribed to an insurance policy covering, in its terms and provisions, its legal liability for injuries caused to participating persons and arising out of this research performed strictly in accordance with the scientific protocol as well as with applicable law and professional standards.

13 STUDY MONITORING AND AUDITING

Monitoring and auditing procedures developed by the sponsor will be followed, in order to comply with GCRP guidelines. On-site checking of the case report forms for completeness and clarity, cross-checking with source documents and clarification of administrative matters will be performed.

13.1 Study monitoring

The study will be monitored by the sponsor. Monitoring will be done by personal visits from a representative of the sponsor (site monitor) who will review the case report forms and source documents. By frequent communications (letter, telephone, and fax), the site monitor will ensure that the investigation is conducted according to protocol design and regulatory requirements.

All unused study materials are to be returned to the sponsor after the clinical phase of the trial has been completed.

13.2 Source data verification and on-site audits

Regulatory authorities, the IEC/IRB, and/or the sponsor's clinical quality assurance group may request access to all source documents, case report forms, and other study documentation for on-site audit or inspection. Direct access to these documents must be guaranteed by the investigator, who must provide support at all times for these activities.

14 DOCUMENTATION AND USE OF STUDY FINDINGS

14.1 Documentation of study findings

A case report form will be provided by the sponsor for each subject.

All protocol-required information collected during the study must be entered by the investigator, or designated representative, in the case report form (except for the Daily Diary Card). Details of case report form completion and correction will be explained to the investigator. If the investigator authorises other persons to make entries in the case report form, the names, positions, signatures, and initials of these persons must be supplied to the sponsor.

The investigator, or designated representative, should complete the case report form pages as soon as possible after information is collected, preferably on the same day that a study subject is seen for an examination, treatment, or any other study procedure. Any outstanding entries must be completed immediately after the final examination. An explanation should be given for all missing data.

The completed case report form must be reviewed and signed by the investigator named in the study protocol or by a designated sub-investigator.

The sponsor will retain the originals of all case report forms. The investigator will retain a copy of all completed case report form pages.

14.2 Use of study findings

All information concerning the product as well as any matter concerning the operation of Hoechst Marion Roussel, such as clinical indications for the drug, its formula, methods of manufacture and other scientific data relating to it, that have been provided by Hoechst Marion Roussel and are unpublished, are confidential and must remain the sole property of Hoechst Marion Roussel. The investigator will agree to use the information only for the purposes of carrying out this study and for no other purpose unless prior written permission from Hoechst Marion Roussel is obtained.

The sponsor has full ownership of the original case report forms completed as part of the study.

By signing the study protocol, the investigator agrees that the results of the study may be used for the purposes of national and international registration, publication, and information for medical and pharmaceutical professionals. If necessary, the authorities will be notified of the investigator's name, address, qualifications, and extent of involvement.

The sponsor will prepare a final report on the study.

The investigator will be required to sign a statement that he or she has read the report and confirms that, to the best of his or her knowledge, it accurately describes the conduct and results of the study.

The findings of the study may be published in a scientific journal or presented at a scientific meeting. Before submitting the results of the study for publication or presentation, the investigator will allow Hoechst Marion Roussel 30 days in which to review and comment on the manuscript.

For any publication manuscript prepared by Hoechst Marion Roussel, Hoechst Marion Roussel reserves the right to select the investigators who will be authors and review the manuscript. Hoechst Marion

Roussel will allow the selected investigators 30 days for full review of the manuscript before publication. It is generally preferable that the results of the multicentre study be published together.

15 STUDY DURATION AND DATES

The duration of this study is expected to be 18 months.

Recruitment initiated	January 2000
Last patient to be randomised	July 2000
End of follow up period	October 2001

16 DECLARATIONS OF SPONSOR AND INVESTIGATOR

16.1 Declaration of sponsor

This study protocol was subject to critical review and has been approved by the appropriate protocol review committee of the sponsor. The information it contains is consistent with:

- The current risk-benefit evaluation of the investigational product
- The moral, ethical, and scientific principles governing clinical research as set out in the Declaration of Helsinki, the ICH Harmonised Tripartite Guideline "Good Clinical Practice: Consolidated Guideline", and the principles of GCP as described in Guidelines for Good Clinical Research Practice (GCRP) in Australia *(or equivalent in participating countries)*.
- The investigator will be supplied with details of any significant or new findings, including adverse events, relating to treatment with the investigational product.

Study manager

Date: ____________ Signature:____

Name (block letters):__________________________________

Biometrician

Date: ____________ Signature:____

Name (block letters):__________________________________

16.2 Declaration of investigator

I have received the following:

- Product Information on the investigational products.

I have been adequately informed about the development of the investigational product. I have read this study protocol and agree that it contains all the information required to conduct the study. I agree to conduct the study as set out in this protocol.

I will not enrol the first subject in the study until I have received approval from the appropriate IEC and until all legal requirements in my country have been fulfilled.

- The study will be conducted in accordance with the moral, ethical, and scientific principles governing clinical research as set out in the Declaration of Helsinki the ICH Harmonised Tripartite Guideline "Good Clinical Practice: Consolidated Guideline", and the principles of GCP as described in Guidelines for Good Clinical Research Practice (GCRP) in Australia *(or equivalent in participating countries).*

I agree to obtain, in the manner described in this study protocol, written informed consent or witnessed verbal informed consent to participate for all subjects enrolled in this study.

I am aware of the requirements for the correct reporting of serious adverse events, and I undertake to document and to report such events as requested.

I agree to supply the sponsor with evidence of current laboratory accreditation, the name and address of the laboratory, and a list of normal values and ranges.

I agree with the use of results of the study for the purposes of national and international registration, publication, and information for medical and pharmaceutical professionals.

I agree to keep all source documents and case report forms as specified in *Section 12.9 Record retention* of this protocol.

I will provide a curriculum vitae before the study starts, which may be submitted to regulatory authorities.

Investigator

Date: ____________ Signature: ____

Name (block letters): ________________________________

17 REFERENCES

1. S Redline. The epidemiology of COPD. In: *Chronic Obstructive Pulmonary Disease.* Ed NS Cherniack. P225-234. WB Saunders. 1991

2. CD Beaty et al. *Chlamydia pneumoniae*, strain TWAR, infection in patients with Chronic Obstructive Pulmonary Disease. *Am Rev Respir Dis* 1991; 141: 1408-1410

3. F Blasi et al. Chlamydia pneumoniae infection in acute exacerbations of COPD. *Eur Respir J.* 1993; 6: 19-22

4. C-C Kuo et al. Demonstration of Chlamydia pneumoniae in atherosclerotic lesions of coronary arteries. *J Infect Dis* 1993; 167: 841-849

5. JB Muhlestein et al. Increased incidence of chlamydia species within the coronary arteries of patients with symptomatic atherosclerotic versus other forms of cardiovascular disease. *J Am Coll Cardiol* 1996; 27: 1555-1561

6. Ramirez JA et al. Isolation of Chlamydia pneumoniae from the coronary artery of a patient with atherosclerosis. *Ann Intern Med* 1996; 125: 979-982

7. LA Jackson et al. Isolation of *Chlamydia pneumoniae* from the carotid endarterectomy specimen. *J Infect Dis* 1997; 176: 292-295

8. L von Hertzen et al. *Chlamydia pneumoniae* infection in patients with chronic obstructive pulmonary disease. *Epidemiol Infect* 1997; 118: 155-164

9. M Leinonen. Pathogenetic mechanisms and epidemiology of *Chlamydia pneumoniae. Eur Heart J* 1993; 14 (Suppl K): 57-61

10. VM Keatings et al. Differences in interleukin-8 and tumour necrosis factor-α in induced sputum from patients with chronic obstructive pulmonary disease and asthma. *Am rev Respir Crit care Med* 1996; 153: 530-534

11. CM Fletcher et al. Value of chemoprophylaxis and chemotherapy in early bronchitis. *BMJ* 1966; I: 1317-1322

12. MR Hammerschlag et al. Persistent infection with *Chlamydia pneumoniae* following acute respiratory illness. *Clin Infect Dis* 1992; 14: 178-182.

13. MR Hammerschlag et al. In vitro activities of five quinolones against Chlamydia pneumoniae. *Antimicrob Agents Chemother* 1992; 36: 682-683

14. PM Robin et al. In vitro activities of oPC-17116, a new quinolone; ofloxacin; and sparfloxacin against *Chlamydia pneumoniae. Antimicrob Agents Chemother* 1994; 38: 1402-1403

15. Allegra L, Konietzko N, Leophonte P, Hosie J, Pauwels R, Guyen JN, Petitpretz P. Comparative safety and efficacy of sparfloxacin in the treatment of acute exacerbations of chronic obstructive pulmonary disease: a double-blind, randomised, parallel, multicentre study. Journal of Antimicrobial Chemotherapy. 37 Suppl A:93-104, 1996.

16. Soler N, Torres A, Ewig S, Gonzalez J, Celis R, El-Ebiary M, Hernandez C, Rodriguez-Roisin R. Bronchial microbial patterns in severe exacerbations of chronic obstructive pulmonary disease (COPD) requiring mechanical ventilation. American Journal of Respiratory and Critical Care Medicine. 157:1498-505, 1998.

17. Van Saene HK, Willems FT, Zweens J. Influence of amoxycillin and cefaclor on the colonization resistance of the oropharynx. Scandinavian Journal of Infectious Diseases – Supplementum. 39:97-9, 1983.

18. Floor M, van Akkeren F, Rozenberg-Arska M, Visser M, Kolsters A, Beumer H, Verhoef J. Effect of loracarbef and amoxicillin on the oropharyngeal and intestinal microflora of patients with bronchitis. Scandinavian Journal of Infectious Diseases. 26:191-7, 1994.

19. Siafakas NM, Vermiere P, Pride NB, et al. Optimal assessment and management of chronic obstructive airways disease (COPD): European Respiratory Society consensus statement. *Eur Respir J* 1995; 8: 1398-420

APPENDIX A

Cockcroft Gault Formula

$$CrCl\ (mL/min) = \frac{(140 - age) \times 1.2 \times \text{Ideal Weight (kg)}}{\text{Serum Creatinine (micromol/L)}} \text{ for males}$$

For females, multiply the resulting value by 0.85

If the ideal weight is not easily calculated, the actual weight is usually satisfactory except in the morbidly obese*. This formula will not accurately predict clearance in patients with rapidly changing renal function.

*Ideal weight for male	= 50 kg + 0.9 kg/each cm over 152 cm (2.3 kg/each inch over 5 feet)
*Ideal weight for female	= 45.5 kg + 0.9 kg/each cm over 152 cm (2.3 kg/each inch over 5 feet)

Reference: Antibiotic Guidelines 9th Edition January 1996 -Victorian Drug Usage Advisory Committee (Australia)

APPENDIX B

Daily Symptom Score - Exacerbations

Variable	0	1	2	3	4
Breathlessness	Not breathless at rest or on exertion	Not breathless at rest but breathless on moderate exertion (e.g. walking quickly)	Not breathless at rest but breathless on mild exertion (e.g. walking on the level)	Not breathless at rest but breathless on minimal exertion (e.g. getting washed)	Breathless at rest
Sputum Production	None	Mild sputum production (i.e. < 30 mL per day)	Moderate Sputum production (i.e. > 30 mL and < 60 mL per day)	Severe sputum production (i.e. > 60 mL per day)	
Sputum Colour	No sputum produced today	Colourless	White/grey	Light yellow	Dark yellow / green

APPENDIX C

Regimen For Administration Of Medicine – Guidelines

COPD Exacerbation
Antibiotic Guidelines 9th Edition January 1996 -Victorian Drug Usage Advisory Committee (Australia) recommends:

"Treatment should be directed towards relief of accompanying airways obstruction. At least half of the patients will have persistent colonisation of the respiratory tract with *Streptococcus pneumoniae, Haemophilus influenzae* and *Moraxella catarrhalis,* a positive sputum culture is not necessarily indicative of infection despite these organisms being responsible for the more severe exacerbations. Antibiotics have been shown to be of benefit only in the subset of patients who present with a more severe infection"

Criteria for antibiotic treatment are:

- Increased cough and dyspnoea; together with
- Increased sputum volume and/or purulence

For treatment, use:

Amoxycillin 500 mg, 8 hourly for 10 – 14 days

If a β-lactamase producing organism is isolated or the clinical response is slow, change to one of the following:

Cefaclor 500 mg orally, 8 hourly for 10 – 14 days

Or

Amoxycillin/potassium clavulanate 500/125 mg orally, 8 hourly for 10 – 14 days

(Oral corticosteroids (e.g. prednisolone 20 – 40 mg daily for 7 days) should be prescribed as required)"

In addition, other beta-lactam antibiotics (e.g. cefuroxime, cephalexin, penicillin), or sulfamethoxazole; trimethoprim may be used to treat COPD exacerbations in this study if deemed appropriate by the investigator.

APPENDIX D

QUALITY OF LIFE – The Chronic Respiratory Disease Index Questionnaire

Guidelines provided – Background Information and Interviewing Tips

First Administration, 7 Point Scale – Interviewer Form + CRQ Response Sheet attached.

APPENDIX E

SPIROMETRY

Respiratory function testing will be performed according to the guidelines of European Respiratory Society (ERS Working Party – Standardised Lung Function Testing – Official Statement of ERS. Eur Respir J 1993; 6 (Suppl 16): 1-100)

APPENDIX F

Diagnosis of *Chlamydia pneumoniae* Infection

Serology (microimmunofluorescence) will be the gold standard for *Chlamydia pneumoniae* infection diagnosis according to the following criterion:

- IgG titre $\geq$ 1:64

Subjects must be IgG positive.

Microimmunofluorescence testing will be performed with a Labsystems kit according to Wang et al. (Am J Ophthalmol 1970;70: 367-374) using a specific antigen (TW-183).

APPENDIX G

Sputum Collection

All subjects will provide spontaneous sputum on the morning of their visit. If the sample is obtained at home (morning of visit) the subject must put the sample in the refrigerator until they depart home to attend their study visit. All samples will be frozen immediately at -70°C in an appropriate freezer at the study site and will be sent by the centre to the central laboratory on a 3 monthly basis. (Samples can be stored for up to one week at -20°C if necessary).

All subjects will provide spontaneous sputum, however, if the investigator assesses that sputum could be obtained by induction with hypertonic saline safely, this is acceptable.

If the subject is having an acute exacerbation at one of the time points scheduled for collection of sputum, sample collection will be deferred for 3 weeks to allow recovery from the acute exacerbation.

The following measurements will be made on the sputum:

- *C. pneumoniae* PCR
- *sIgA*

APPENDIX H

Laboratory Data

Local laboratories will be used to assess the laboratory parameters listed below

Haematology	Biochemistry
• Haemoglobin	• AST
• Haematocrit	• ALT
• Red blood cells	• Gamma-glutamyl transferase
• WBC (total and differential)	• Lactic dehydrogenase
• Monocytes	• Alkaline phosphatase
• Platelet count	• Creatinine
• PT ratio /INR (if appropriate)	• Total bilirubin
• Digoxin levels (if appropriate)	• Glucose
• Theophylline levels (if appropriate)	• Albumin
	• Urea
	• Amylase or Lipase
	• Calcium
	• Sodium
	• Potassium
	• Chloride

APPENDIX I

Blood Specimen Collection and Storage

- Serology blood should be collected at each relevant visit to enable 4 mL of serum to be separated
- 2 samples of 2 mL serum should be stored in microtubes (e.g. Eppendorf safe-twist) and labelled with appropriate label including patient initials, patient number, visit number and date.
- Samples for assessing the inclusion criterion (IgG titre≥ 1:64) will be sent immediately to the central laboratory.
- Other samples should be stored in -70°C until transported to central laboratory (3 monthly). Can be stored for up to one week in - 20°C (monitored freezer), if -70°C unavailable. Should then be transported to central laboratory.

The antibody detection will be performed with a Labsystems® kit for *Chlamydia pneumoniae* by means of microimmunofluorescence. The test will be performed according to the manufacturer's instructions.

Laboratories conducting serology testing

Australia

Dorevitch Laboratory Services

New Zealand

Waikato Hospital

APPENDIX J

Microbiological evaluation of sputum

Sputum will be cultured at selected sites to determine the effect of long term broad spectrum antibiotic therapy on the resident flora of sputum.

The methods may vary at different sites.
